# A C‐Nucleoside Analogue of Cordycepin With High Metabolic Stability and Potent Anti‐Psoriatic Activity via Microneedle Delivery

**DOI:** 10.1002/advs.77012

**Published:** 2026-08-05

**Authors:** Wenfang Pan, Xinyue Shao, Yuanchen Zhong, Xujie Sun, Lixuan Yin, Zongyan He, Tianqun Lang, Yuanchao Xie

**Affiliations:** ^1^ Lingang Laboratory Shanghai China; ^2^ School of Physical Science and Technology ShanghaiTech University Shanghai China; ^3^ School of Life Science and Technology ShanghaiTech University Shanghai China

**Keywords:** anti‐inflammation, C‐nucleoside, cordycepin, immunomodulation, microneedle, psoriasis, topical treatment

## Abstract

Psoriasis is a chronic inflammatory disorder characterized by immune dysregulation and epidermal hyperplasia. Cordycepin, a natural adenosine analogue from traditional Chinese medicine *Cordyceps militaris*, possesses broad anti‑inflammatory and immunomodulatory properties, but its poor metabolic stability limits clinical use. Herein, 22 cordycepin derivatives for anti‐psoriasis were designed and synthesized. Among them, **CPD3a**, featuring a stable C─C glycosidic bond, exhibited potent anti‑inflammatory activity, high metabolic stability, and high resistance to in vivo metabolic deamination. **CPD3a** was formulated into a microneedle array (**CPD3a**‐MN) for the topical treatment of psoriasis. In a psoriasis‐like mouse model, **CPD3a**‐MN exhibited potent, dose‐dependent efficacy, significantly reducing pathological symptoms, ameliorating epidermal hyperplasia, and suppressing systemic inflammation. Meanwhile, **CPD3a**‐MN rebalanced systemic immunity by suppressing splenic neutrophils and inflammatory dendritic cells while increasing regulatory T cells and M2 macrophages. Activation of adenosine monophosphate‐activated protein kinase (AMPK)/nuclear factor erythroid 2‐related factor 2 (NRF2) pathway and a marked antioxidant effect were also observed following **CPD3a**‐MN treatment. Transcriptomic analysis demonstrated that **CPD3a**‐MN modulated the psoriatic transcriptome, suppressing interleukin‐17 (IL‐17)/nuclear factor‐kappa B (NF‐κB)‐driven inflammatory networks and concurrently activating genes involved in keratinocyte differentiation and epidermal barrier restoration. Moreover, **CPD3a**‐MN was well‑tolerated, with good biocompatibility. Collectively, **CPD3a** represents a superior therapeutic alternative to cordycepin for topical psoriasis treatment.

## Graphical abstract

This work identified **CPD3a** as a potent, stable C‐nucleoside cordycepin derivative. When formulated into microneedle array for topical treatment, it ameliorated psoriasis by rebalancing immunity and enhancing antioxidant defenses.

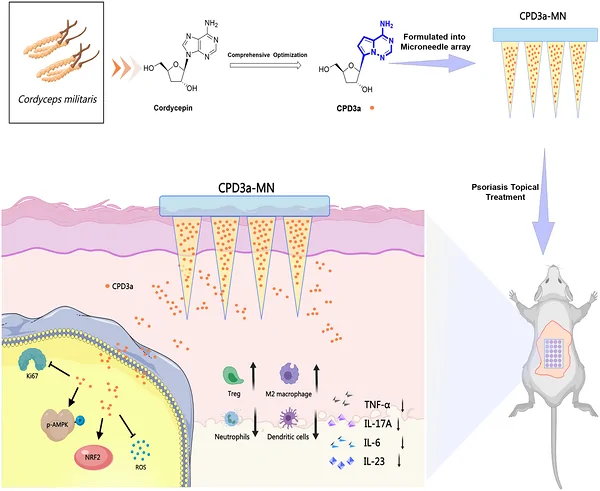

## 1 Introduction

Cordycepin (3′‐deoxyadenosine) is an adenosine analogue isolated from *Cordyceps militaris—*a parasitic fungus highly valued in traditional Chinese medicine [1]. It possesses multiple biological activities, including anti‐neoplastic, antibiotic, antiviral, and antioxidant effects [2, 3, 4, 5, 6]. These attributed pharmacological properties have driven extensive scientific investigation into cordycepin and led to its utilization in various health‐promoting products, functional foods, and natural medicinal preparations. In recent years, growing evidence has highlighted the therapeutic potentials of cordycepin on inflammation and immune‐related diseases [7, 8, 9, 10]. Among chronic inflammatory diseases, psoriasis represents a significant global health burden, affecting an estimated 2∼3% of the population worldwide [11]. Psoriasis is clinically defined by cutaneous manifestations such as scaly plaques, erythema, and intense pruritus, driving a continuous need for effective therapeutic agents [12, 13]. Notably, cordycepin has been shown to inhibit IL‑17A‑induced proliferation, downregulate proliferating cell nuclear antigen (PCNA) and Ki‑67, restore apoptosis by modulating Bcl2‐associated X protein (BAX), B‐cell lymphoma‐2 (Bcl‑2), and p53 in keratinocytes, and ameliorate psoriasiform skin lesions in imiquimod (IMQ)‐induced murine models [14], suggesting its potential as an anti‐psoriatic agent.

Despite this interest, the clinical development of cordycepin has been hindered by its inherent pharmacokinetic limitations. A major challenge is its metabolic instability, as cordycepin is rapidly deaminated by adenosine deaminase (ADA) to the inactive metabolite 3′‐deoxyinosine to diminish its efficacy [15]. Although structural optimizations of cordycepin have been explored, only marginal improvements in the pharmacokinetic (PK) properties have been achieved, often at the expense of its biological activities [16, 17, 18, 19].

In the present study, a library of **22** novel cordycepin derivatives was designed and synthesized, which was categorized into two distinct structural classes based on the glycosidic bond (C─N bond or C─C bond). In vitro screening identified four compounds with strong anti‐inflammatory activities in lipopolysaccharide (LPS)‑stimulated THP‑1 macrophages. Among them, a C‐nucleoside analog of cordycepin, **CPD3a**, demonstrated remarkable in vitro metabolic stability and excellent PK properties in mice. **CPD3a** topically administered via a microneedle delivery system (**CPD3a**‐MN) exhibited potent, dose‐dependent anti‐psoriatic efficacy in an IMQ‐induced psoriasis‐like mouse model (Scheme 1). In addition, **CPD3a**‐MN modulated systemic immunity by suppressing splenic neutrophils and inflammatory dendritic cells (DCs) while increasing regulatory T cells (Tregs) and M2 macrophages. Transcriptomic analysis indicated that **CPD3a** exerted a coordinated intervention on multiple pathological pathways, positioning it as a potential approach for the treatment of psoriasis.

**SCHEME 1:**
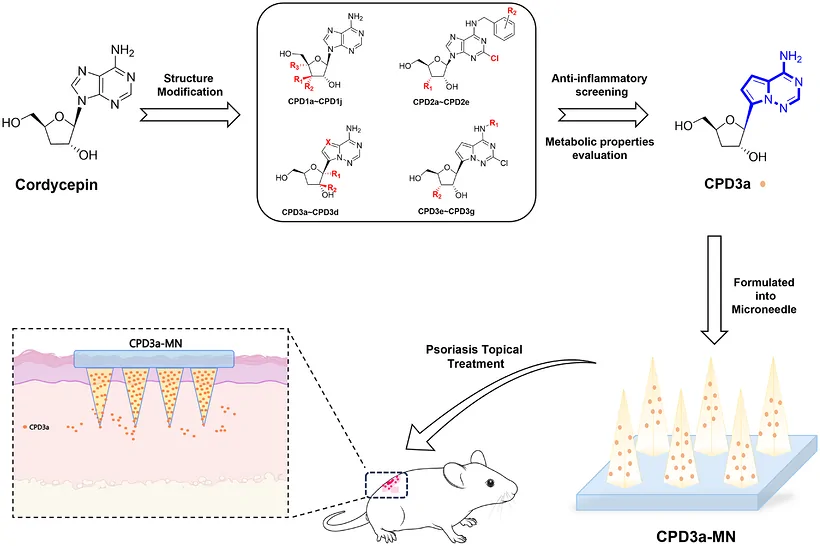
Schematic illustration of the design and synthesis of cordycepin derivatives, formulation of **CPD3a**‐MN, and therapeutic efficacy in IMQ‐induced psoriasis mouse model.

## 2 Results and Discussion

### 2.1 In Vitro Anti‐Inflammatory Evaluation of Cordycepin Derivatives

Tumor necrosis factor (TNF)‐α is a typical proinflammatory cytokine, critically involved in the pathogenesis of psoriasis, as well as other inflammatory and autoimmune diseases [20]. Therefore, an initial screening of cordycepin derivatives was performed by assessing inhibition of TNF‐α release in LPS‐induced THP‐1 cells at concentrations of 25 and 2.5 µm with dexamenthasone (10 µm) as the positive control. Cytotoxicity was evaluated in parallel by measuring cell viability at 25 µm (Table S1).

Typically, structural modifications of nucleoside analogs are performed at the ribose moiety, the nucleobase, or the glycosidic bond [21, 22]. The designed cordycepin derivatives were categorized into two types. Type I (**CPD1a∼CPD2e**) featured modifications at the sugar or the base moiety of cordycepin while preserving the natural C─N glycosidic bond. Type II (**CPD3a∼CPD3g**) belonged to C‐nucleosides, featuring non‐natural purine‐like bases that were linked to the sugar moiety via a stable C─C glycosidic bond. The initial structure‐ activity relationship (SAR) studies focused on the ribose moiety. Cordycepin lacks the 3′‐hydroxyl group, which is the only structural difference from adenosine and is likely crucial to its biological activities. Unsurprisingly, all five compounds bearing an *α*‐methoxyl (**CPD1a**), a *β*‐hydroxyl (**CPD1b**), a *β*‐fluoro (**CPD1c**), a *β*‐methyl‐*α*‐hydroxyl (**CPD1d**), or a *β*‐ethynyl*‐α*‐hydroxyl (**CPD1e**) group at the 3′‐position displayed diminished or abolished inhibition activity on TNF‐α release (Figure 1B). In the same system, cordycepin was found to be a mild inhibitor with an inhibition rate of 31% at 25 µm and negligible activity at 2.5 µm. Regarding the 4′‐substituted compounds, **CPD1f∼1j**—respectively bearing a hydroxylmethyl, methyl, fluoromethyl, chloromethyl, and cyno group—showed almost no activity. These results suggested that substitutions, even small groups like methyl or fuoro, were not tolerated at the 3′ or 4′ position on the cordycepin scaffold. Recently, it was reported that some cordycepin derivatives bearing a C2‐chloro substituent and a C6‐benzylamino group demonstrated anti‐hepatic fibrosis activities [23]. In the present study, this kind of compounds showed potent anti‐inflammatory activities exemplified by compound **CPD2c** that possessed a C2‐chlorine and a C6‐2‐chlorobenzylamino group on the purine base moiety. **CPD2c** was significantly more active than cordycepin, with inhibition rates of 80.0% at 25 µm and 31.3% at 2.5 µm on TNF‐α secretion, despite being slightly less effective than dexamenthasone. **CPD2d** and **CPD2e** with an additional substitution (4‐chloro and 4‐fluoro, respectively) on the benzene ring of the C6‐benzylamino group, exerted a similar anti‐inflammatory effect as that of **CPD2c**. However, incorporating a 3′‐*β*‐hydroxyl group (**CPD2a**) or removing the chloro atom (**CPD2b**) from the benzene ring of **CPD2c** resulted in markedly reduced activity.

**FIGURE 1:**
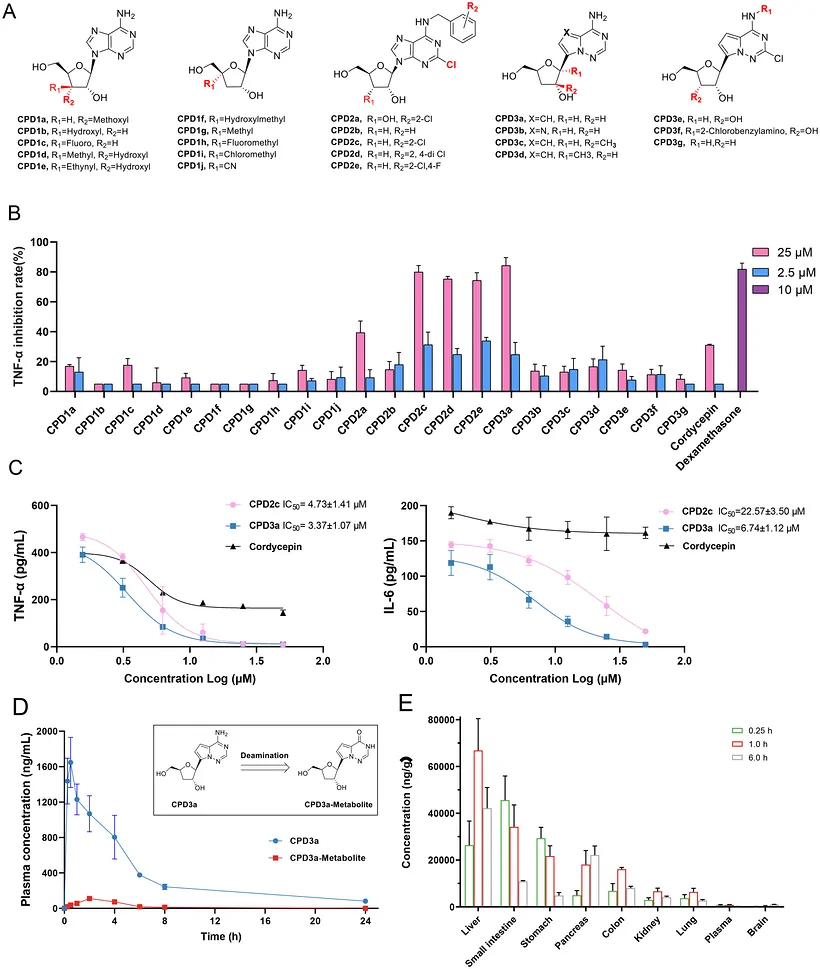
In vitro anti‐inflammatory activity and PK properties. (A) Structures of cordycepin derivatives in the present study; (B) Inhibition of TNF‑α production in LPS‑stimulated THP‑1 macrophages by cordycepin derivatives at 25 and 2.5 µm, measured by enzyme‐linked immunosorbent assay (ELISA). Dexamethasone served as a positive control; (C) IC_50_ of the selected compounds against TNF‐α and IL‐6 secretion; (D) Mean plasma concentrations of **CPD3a** and the metabolite (**CPD3a‐M**) in mice at different time points following a single oral dose of 25 mg/kg **CPD3a**; (E) Tissue distribution of **CPD3a** in mice following a single oral dose of 15 mg/kg. Data were shown as the mean ± SD. For each group, *n* = 3 independent samples in (B–E).

C─C glycosidic bond is resistant to cleavage by nucleoside phosphorylases, and frequently utilized in the modification of nucleoside analogs [24, 25]. Compound **CPD3a,** which contained an adenine‐like pyrrolotriazine base linked to 3'‐deoxyribose through a C─C glycosidic bond, closely resembled cordycepin in structure. Surprisingly, it demonstrated potent activity in inhibiting TNF‐α release, with inhibition rates of 84.3% at 25 µm and 24.7% at 2.5 µm. Minor structural modifications of **CPD3a** were therefore carried out. These included replacing the C7 carbon atom with a nitrogen atom (**CPD3b**), introducing a 2′‐methyl group (**CPD3c**), a 1′‐methyl group (**CPD3d**), a C2‐chloro substitution (**CPD3f**), or a 3′‐hydroxyl‐C2‐chloro substitution (**CPD3g**). However, all these modifications resulted in significantly decreased activity. An additional compound **CPD3f**, bearing a 2‐chlorobenzylamino group at the C6 position, was also designed but exhibited no anti‐inflammatory activity. The half‐maximal inhibitory concentrations (IC_50_) of **CPD2c** and **CPD3a** against proinflammatory cytokine release were then determined. As shown in Figure 1C, **CPD3a** exhibited IC_50_ values of 3.37±1.07 µm for TNF‐α and 6.74±1.12 µm for IL‐6. **CPD2c** showed comparable potency, with IC_50_ values of 4.73 ± 1.41 µm(TNF‐α) and 22.57 ± 3.50 µm (IL‐6). Both compounds were significantly more potent than cordycepin and presented no cytotoxicity at the tested concentrations (Table S2).

### 2.2 In Vitro Metabolic Stability and In Vivo PK Study

The metabolic stability of **CPD2c**, **CPD3a,** and cordycepin was assessed in liver microsomes. In mouse liver microsomes, both **CPD2c** and cordycepin were rapidly metabolized, with short half‐lives (T_1/2_ = 18.2 and 15.5 min, respectively, Table S3). In human liver microsomes, **CPD2c** demonstrated moderate stability (T_1/2_ = 52.3 min), which was notably better than that of cordycepin (T_1/2_ = 27.2 min). In stark contrast, **CPD3a** exhibited exceptional stability in both species, with half‐lives exceeding 17 h (human: 1145.6 min; mouse: 1059.8 min) and very low intrinsic clearance (human CL_int_ = 1.5 mL/min/kg; mouse CLint = 5.2 mL/min/kg, Table S3), confirming its high resistance to hepatic microsomal metabolism.

Given that liver microsomes lack key cytosolic enzymes involved in purine nucleoside metabolism (e.g., nucleoside phosphorylases and adenosine deaminase), the in vitro metabolic profile may not fully recapitulate the in vivo situation. Thus, the in vivo PK properties of **CPD3a** in mice were evaluated. As shown in Table S4, following intravenous (IV) administration, **CPD3a** demonstrated a long elimination half‐life (11.5 h), low systemic clearance (32.7 mL/min/kg), and a high apparent volume of distribution at steady state (Vss = 22.5 L/kg), indicative of marked metabolic stability and extensive tissue distribution. The plasma concentrations of its speculated ADA‐mediated deamination metabolite, **CPD3a‐M**, were not quantifiable at most time points. After oral administration (PO), **CPD3a** achieved high bioavailability (81.8%), reflecting remarkably efficient absorption. Notably, the metabolite **CPD3a‐M** was detected in plasma, but its maximal concentration was approximately 14‐fold lower than that of the parent compound (111 vs 1647 ng/mL, Figure 1D and Table S4). This profile suggested that **CPD3a‐M** was formed primarily via first‐pass intestinal and/or hepatic metabolism. In comparison, cordycepin demonstrated negligible systemic exposure following intravenous or oral administration, with only its deaminated metabolite 3′‑deoxyinosine, detected as the circulating analyte (Table S5). Collectively, the data characterize **CPD3a** as a metabolically stable, orally bioavailable, and long‐acting anti‐inflammatory agent.

Moreover, the tissue distribution of orally administered **CPD3a** in mice was investigated. Consistent with the high value of Vss in the PK study, **CPD3a** was found to be widely distributed in many tissues (Figure 1E), with maximum concentrations detected in the liver, followed by small intestine, stomach, pancreas, kidney, and lung. Importantly, the plasma concentration of **CPD3a** was substantially lower than that in most tissues and was comparable to the level detected in the brain.

### 2.3 Preparation and Characterization of CDP3a‐Loaded Microneedles

Considering **CPD3a**’s high accumulation in the liver and gastrointestinal tract following oral administration, topical administration represents a preferred route for evaluating its in vivo anti‐psoriasis efficacy. However, psoriatic skin lesions, characterized by excessive keratinization, significantly impede drug penetration [13]. In this context, microneedle (MN) technology offers a compelling strategy to bypass the skin barrier and enable localized delivery. To date, various MN delivery systems have been developed for skin disease treatment [26, 27, 28, 29, 30].

To enable effective topical treatment of psoriasis, **CPD3a** was formulated into a dissolving microneedle array (**CPD3a**‑MN) for localized intradermal delivery. The microneedles were prepared with the micro‐molding method as illustrated in Figure 2A. A **CPD3a**‐polyvinylpyrrolidone (PVP) solution was first filled into a polydimethylsiloxane (PDMS) mold. A backing layer of polyvinyl alcohol‐PVP (PVA‐PVP) was then cast over the mold. After drying, the solidified, layered patch was demolded to yield the final microneedle array. Digital equipment image revealed intact needle bodies of **CPD3a**‑MN (Figure 2B). Scanning electron microscope (SEM) images also demonstrated that the microneedle arrays possessed a well‐defined, regular quadrangular pyramid geometry and uniform array arrangement (Figure 2C). Each needle featured a sharp tip with a height of approximately 600 µm and a square base with a side length of 300 µm. Meanwhile, all needles were arranged in a uniform 10 × 10 array, with an inter‐needle spacing of about 550 µm (Figure 2C). Representative confocal laser scanning microscopy (CLSM) images showed uniform distribution of the fluorescent tracer throughout the tips, which served as an indicator of the homogeneous dispersion of **CPD3a** within the polymer matrix (Figure 2D). Similarly, fluorescein isothiocyanate (FITC)‐labeled **CPD3a** was prepared to assess its distribution at the needle tip (FITC‐**CPD3a**‐MN). CLSM images revealed uniform green fluorescence across the needle tip, indicating uniform distribution without agglomeration (Figure S1). The ultraviolet (UV) absorption wavelength of **CPD3a** was 244 nm (Figure S2A). The content of **CPD3a** in the microneedles was determined by high‐performance liquid chromatography (HPLC), and the content of **CPD3a** in MN tip was 226.90 ± 5.93 µg (Figure S2B,C). Meanwhile, the content of **CPD3a** in the MN baseplate was 3229.22 ± 170.82 µg. After **CPD3a**‐MN application to the skin for 1 h, the drug content in the skin tissue was determined to be 179.82 ± 0.58 µg. The mechanical strength of the **CPD3a**‐MN was evaluated to verify its ability to penetrate the skin. Under compression testing, the array exhibited a maximum load of 23.454 N at a displacement of 500 µm (Figure 2E). The result corresponded to an approximate force of 0.23 N per needle tip, confirming that the **CPD3a**‐MN patch possessed sufficient mechanical integrity to effectively breach the stratum corneum for intradermal delivery [31]. Furthermore, the skin penetration capability of **CPD3a**‐MN was confirmed through visual documentation and histological examination, as presented in Figure 2F. The skin pores completely disappeared in 25 min after **CPD3a**‐MN application, and there was no significant skin irritation, such as redness, swelling, or bleeding, or skin corrosion, demonstrating the favorable skin safety of **CPD3a**‐MN (Figure S3, Table S6). Following insertion of the **CPD3a**‐MN patch, an array of micro‐perforations in the murine dorsal skin was evident upon staining with trypan blue (Figure 2F). Hematoxylin and eosin (H&E) staining of treated skin revealed the formation of microchannels with a depth of approximately 640 µm, corresponding to the microneedle height. In addition, CLSM images of skin tissues at various depths following FITC‐**CPD3a**‐MN treatment revealed green fluorescent FITC‐**CPD3a** at all depth planes, with vertical diffusion reaching up to 200 µm (Figure S4). Hence, **CPD3a**‐MN successfully penetrated the skin, rapidly released the drug from the tip, and effectively delivered **CPD3a** into deep skin layers. Regarding the release profile, the cumulative release of **CPD3a** from the MN patch gradually increased, reaching more than 68.7% within 4 h, and 95.4% by 12 h (Figure 2G). The Franz diffusion cell experiment results indicated that the cumulative permeation amount of the **CPD3a** from the **CPD3a**‐MN increased over time during the 6 h period, and the cumulative permeation rate reached approximately 34% (Figure S5), which supports the favorable transdermal permeation behavior of soluble microneedles. Additionally, the sequential images of **CPD3a**‑MN patch pierced into the mouse skin were recorded by SEM to verify the degradation property of microneedle patch in vivo. The SEM images distinctly showed a gradual decrease in the height of the needle tip during the 30 min duration, indicating that soluble **CPD3a**‐MN could be effectively degraded in the skin interstitial fluid to release the drug (Figure 2H). Moreover, the cytotoxicity of **CPD3a**, PVP and **CPD3a** combined with PVP (**CPD3a**+PVP) was evaluated in two skin‑relevant cell lines, HaCaT and NIH3T3. **CPD3a** alone and **CPD3a**+PVP showed comparable cytotoxicity in both cell lines, with CC_50_ values of >100 µm (HaCaT) and ∼100 µm (NIH3T3). PVP alone did not affect cell viability at any concentration tested, confirming its biocompatibility as a microneedle matrix material (Figure S6). In summary, **CPD3a**‑MN possesses sufficient mechanical strength to overcome the stratum corneum barrier and effectively deliver the drug to therapeutically relevant depth in the skin.

**FIGURE 2:**
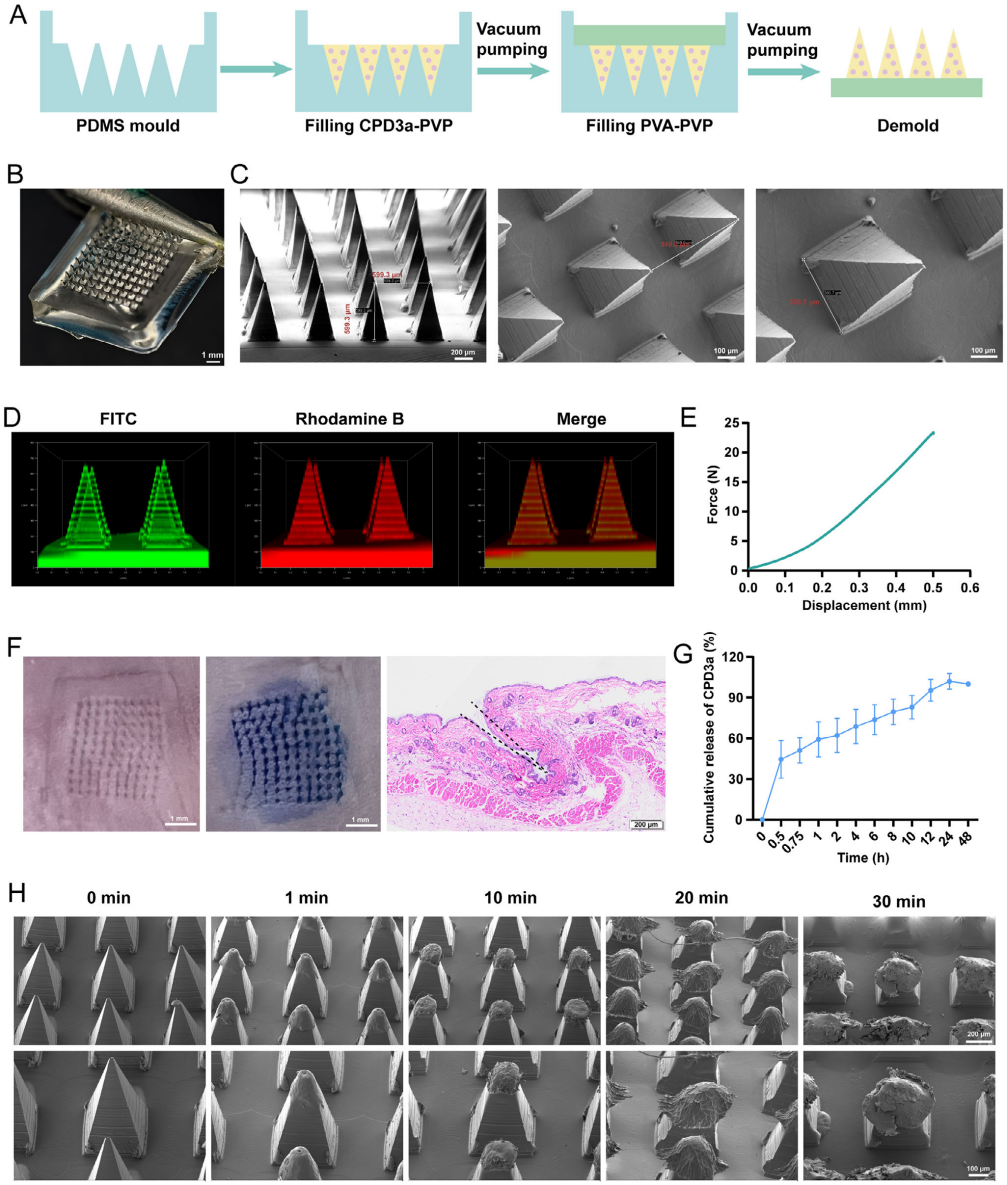
Characterization of **CPD3a**‐MN. (A) Schematic illustration of **CPD3a**‐MN fabrication. (B) Digital photograph of **CPD3a**‐MN (Scale bar: 1 mm). (C) Scanning electron microscope (SEM) images of **CPD3a**‐MN (Scale bar: 200 µm; Scale bar:100 µm). (D) Representative CLSM images and 3D construct of MN labeled with FITC (green) and Rhodamine B (red). (E) Mechanical properties of **CPD3a**‐MN. (F) Representative photograph (Scale bar: 1 mm) and H&E staining image (Scale bar: 200 µm) of the mouse skin after **CPD3a**‐MN insertion. (G) Cumulative release curve of **CPD3a** from the MN in vitro. (H) Morphology of the needle tip of **CPD3a**‐MN in mouse skin at different time points (Scale bar: 200 µm; Scale bar:100 µm). Data were shown as the mean ± SD. For each group, *n* = 3 independent samples in (G).

### 2.4 CPD3a‑MN Dose‑Dependently Ameliorates Psoriasis in Mouse

To evaluate the in vivo therapeutic efficacy of **CPD3a**‐MN, IMQ‐induced psoriasis‐like mouse model was established over a 7‐day treatment period, as outlined in Figure 3A. Disease progression was monitored daily according to the Psoriasis Area and Severity Index (PASI) score, and skin tissues were collected at the endpoint for histopathological analysis.

**FIGURE 3:**
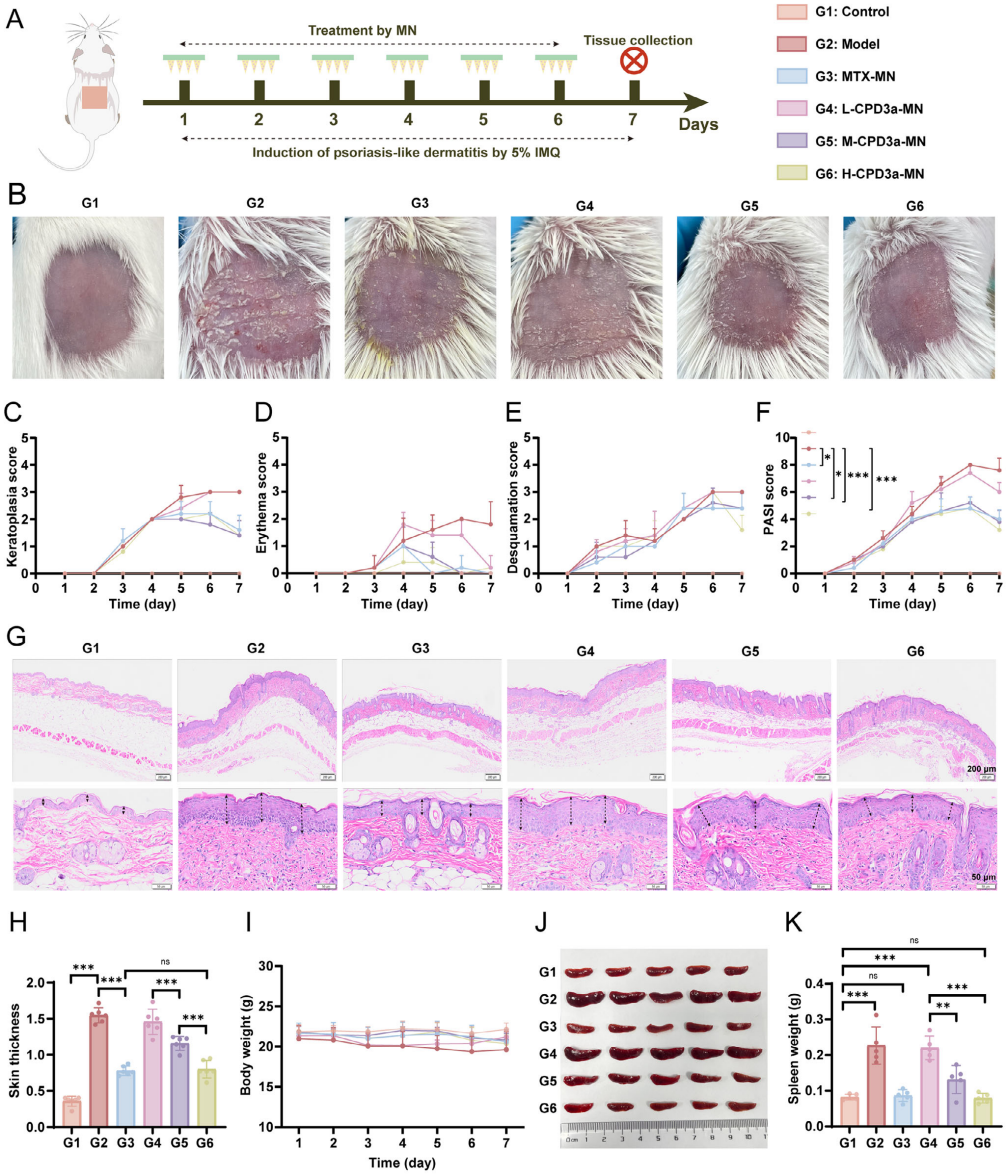
In vivo therapeutic effect of **CPD3a**‐MN. (A) Schematic illustration of the treatment schedule of **CPD3a**‐MN on 5% IMQ‐induced psoriasis‐like mice. (B) Representative optical images of mice dorsal lesions after different treatments on day 7. PASI score of (C) keratoplasia, (D) erythema, (E) desquamation, and (F) total score from day 0 to 7. (G) H&E staining images and their local magnifications of the mice's dorsal skin after different treatments on day 7 (Scale bar: 200 µm; Scale bar: 50 µm). The black dashed double‐headed arrows represented the thickness of the epidermis. (H) The statistical analysis of epidermal thickness in each group. (I) Body weight of mice during the treatment. (J) Photographs of the spleen tissue at the endpoint. (K) The weight of the spleen tissue at the endpoint. Data were shown as the mean ± SD. For each group, *n* = 5 independent samples in (C‐F, I‐K); *n* = 3 independent samples in (G), *n* = 6 independent samples in (H). ^*^ *p* < 0.05, ^**^ *p* < 0.01, ^***^ *p* < 0.001.

Treatment with IMQ resulted in the rapid development of severe psoriasiform dermatitis, visually characterized by pronounced scaling, intense erythema, and marked dorsal skin thickening (Figure 3B). Daily scoring revealed that treatment with **CPD3a**‐MN significantly suppressed the progression of all major psoriatic features in a clear dose‐dependent manner. Specifically, high doses of **CPD3a**‐MN markedly alleviated keratoplasty (Figure 3C), erythema (Figure 3D), and desquamation (Figure 3E), resulting in a much lower total PASI score compared to the model group (Figure 3F). Similarly, PASI scores in the methotrexate (MTX)‐MN group (positive control) were also lower after day 6. H&E staining of skin sections provided histological confirmation of the treatment effects of **CPD3a**. **CPD3a**‐MN treatment ameliorated the characteristic epidermal hyperplasia, significantly reducing the thickness of the mice's dorsal skin in the high dose group (Figure 3G,H). Importantly, the treatment exhibited undetectable systemic toxicity, as evidenced by stable body weights across all treatment groups throughout the experimental timeline (Figure 3I). Furthermore, IMQ induction triggered distinct splenomegaly, reflected in increased spleen size and weight, a hallmark of systemic immune activation (Figure 3J). The splenomegaly caused by IMQ was notably and dose‐dependently reversed by **CPD3a**‐MN treatment (Figure 3J,K), indicating a potent systemic attenuation of the hyperimmune response.

### 2.5 CPD3a‑MN Ameliorates Psoriasis by Modulation of Inflammation and Antioxidant Pathways

To investigate the mechanisms underlying the therapeutic effect of **CPD3a**, the molecular hallmarks of psoriasis were first examined. The primary pathological features of psoriatic lesions included abnormal keratinocyte proliferation and sustained inflammation [12, 13]. Immunohistochemical analysis of Ki67, a marker of proliferating cells [32], revealed a significant reduction in keratinocyte proliferation across all **CPD3a**‐treated groups compared to the model group, with the H‐**CPD3a**‐MN group showing the most pronounced inhibition (Figure 4A). As the central driver of psoriatic pathology, cytokine IL‐17A is mainly produced by Th17 cells [33]. Upon binding to keratinocyte receptors, IL‐17A triggers a proinflammatory cascade that further stimulates keratinocyte hyperproliferation and recruits immune cells to the skin [34]. Treatment with **CPD3a** efficiently downregulated IL‐17A expression (Figure 4A), disrupting the pathogenic cycle. Consistent with the immunohistochemical results, levels of key inflammatory mediators—IL‐17A, IL‐23, IL‐6, and TNF‐α—were also reduced in both mouse serum and spleen tissue following **CPD3a** treatment (Figure 4B–I).

**FIGURE 4:**
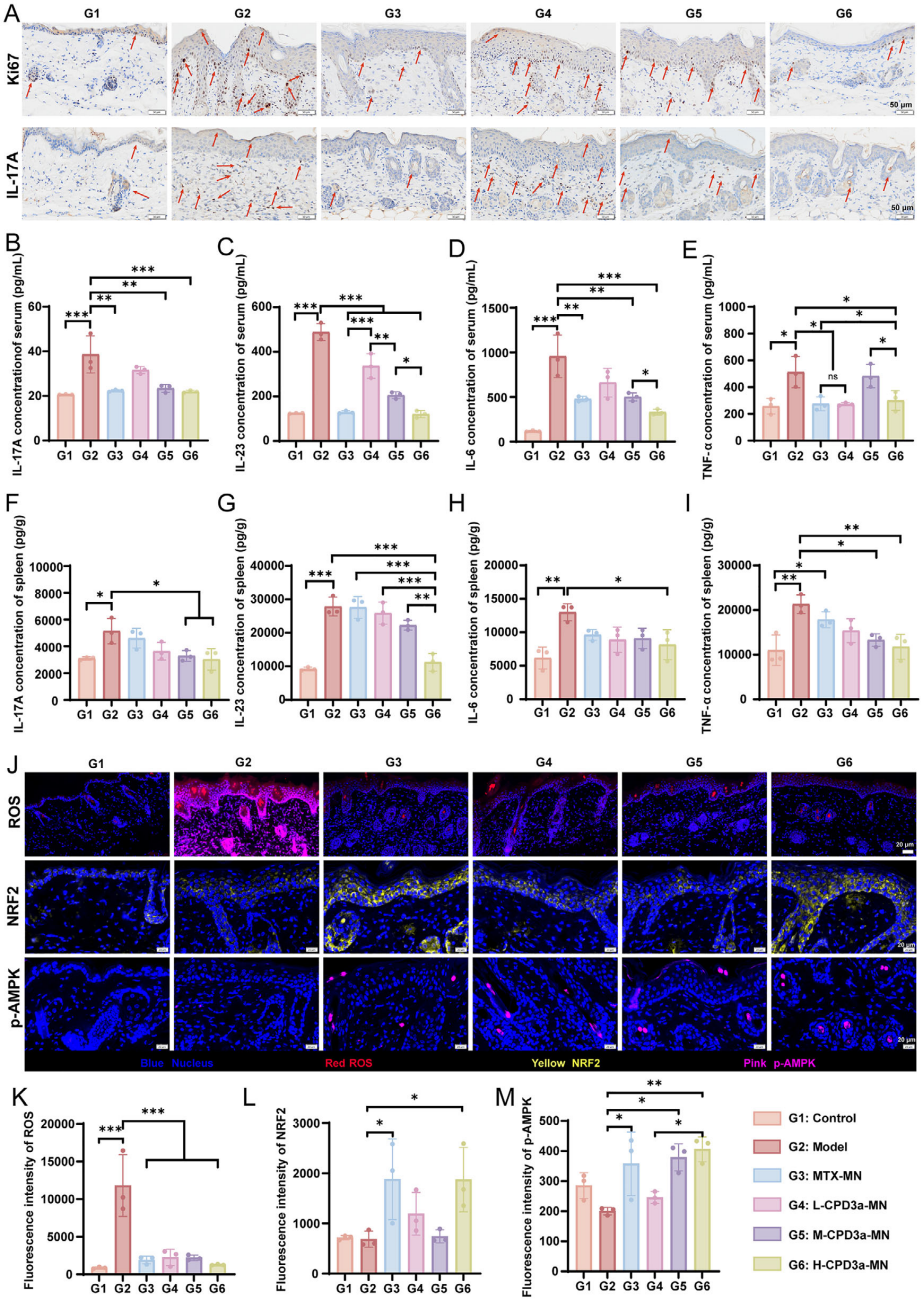
In vivo anti‐inflammation mechanism of CPD3a‐MN. (A) The immunohistochemical staining of Ki67 and IL‐17A of mice dorsal skin lesions (Scale bar: 50 µm). The red arrows represented the expression of Ki67 and IL‐17A. The concentration of (B) IL‐17A, (C) IL‐23, (D) IL‐6, (E) TNF‐α in serum from psoriasis‐like mice after treatment. The concentration of (F) IL‐17A, (G) IL‐23, (H) IL‐6, (I) TNF‐α in spleen tissues from psoriasis‐like mice after treatment. (J) The immunofluorescence section of ROS, NRF2, and p‐AMPK of mice dorsal skin lesions (Scale bar: 20 µm). (K–M) The statistical analysis of fluorescence intensity of ROS, NRF2, and p‐AMPK from psoriasis‐like mice after treatment. Data were shown as the mean ± SD. For each group, *n* = 3 independent samples in (B–I, K–M). ^*^ *p* < 0.05, ^**^ *p* < 0.01, ^***^ *p* < 0.001.

Given the reported role of reactive oxygen species (ROS) in psoriasis development [35], the effect of **CPD3a** on oxidative stress was further investigated. While IMQ induced substantial epidermal ROS overproduction, **CPD3a**‐MN treatment markedly reduced ROS accumulation (Figure 4J,K). The transcription factor nuclear factor erythroid 2‐related factor 2 (NRF2), a key regulator of antioxidant responses, is decreased in psoriatic skin [36]. Immunohistochemical and immunofluorescence staining revealed that **CPD3a** treatment increased NRF2 expression (Figure 4J,L). Furthermore, phosphorylation of AMPK (p‐AMPK) was elevated with **CPD3a** treatment (Figure 4J,M). A cellular thermal shift assay (CETSA) in HaCaT cells indicated binding of **CPD3a** to AMPK, as evidenced by the thermal stabilization of AMPK upon **CPD3a** treatment (Figure S7) [37, 38, 39]. Since AMPK‐mediated phosphorylation of NRF2 could promote NRF2 nuclear translocation and activation of antioxidant gene expression [40], these findings suggest that **CPD3a** might enhance cellular antioxidant defenses via the AMPK–NRF2 pathway.

### 2.6 CPD3a‐MN Restores Systemic Immune Homeostasis in Psoriasis

Given that **CPD3a**‐MN treatment maintained normal spleen size and reduced key inflammatory cytokines of IMQ‐treated mice with systemic immune activation, the modulatory effects of **CPD3a**‐MN on the immune cell composition in spleen tissues were further investigated. To test the specific immune cell subsets alterations, flow cytometric analysis was performed on spleen tissues.

As shown in Figure 5, IMQ treatment induced a significant expansion of multiple innate and adaptive immune cell populations in the spleen. Among them, neutrophils and inflammatory DCs increased obviously—cell types that play essential roles in psoriatic inflammation through antigen presentation and pro‐inflammatory cytokine production [41, 42]. In contrast, the proportions of immunosuppressive cell types, specifically Tregs and M2 macrophages [43, 44], were markedly decreased, indicating a disruption in regulatory feedback mechanisms and a shift toward a hyper‐inflammatory state (Figure 5A,B). Simultaneously, high‐dose **CPD3a**‐MN treatment further elevated the proportion of Tregs by 1.45‐fold and promoted macrophage polarization toward the M2 phenotype by 5.82‐fold, thereby enhancing immune tolerance (Figure 5E,F). Treatment with **CPD3a**‐MN produced a distinct immunomodulatory profile. It effectively suppressed the IMQ‐induced expansion of pro‐inflammatory DCs and neutrophils (Figure 5C,D). Compared with the model group, the proportion of DCs and neutrophils subsets in the H‐**CPD3a**‐MN group decreased by 2.02‐fold and 1.69‐fold, respectively (Figure 5G,H). These findings highlight the role of **CPD3a**‐MN in restoring immune homeostasis in the spleens of psoriasis‐like mice.

**FIGURE 5:**
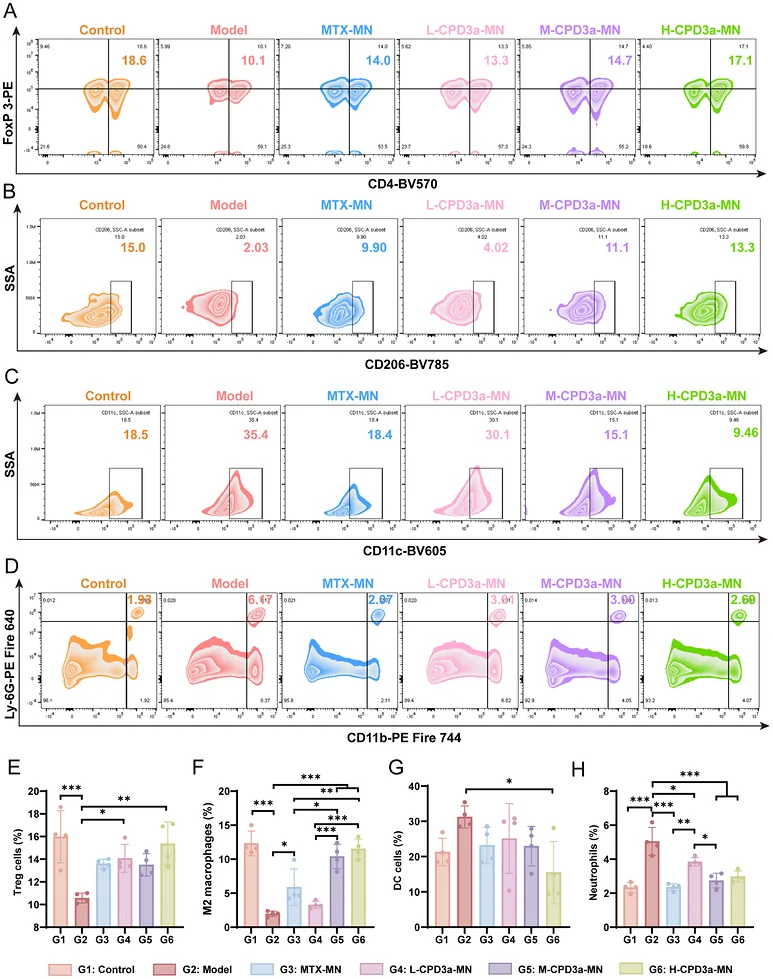
Evaluation of the immune cells in the spleen after **CPD3a**‐MN treatment. Representative flow cytometry images of (A) Treg (CD4 ^+^ Foxp3 ^+^), (B) M2 macrophages (CD206^+^), (C) DCs (CD11c ^+^), and (D) neutrophils (CD11b ^+^ Ly6G ^+^). The statistical analysis of immune cell subsets, including (E) Treg, (F) M2 macrophages, (G) DCs, and (H) neutrophils. Data were shown as the mean ± SD. For each group, *n* = 4 independent samples in (E‐H). ^*^ *p* < 0.05, ^**^ *p* < 0.01, ^***^ *p* < 0.001.

### 2.7 CPD3a‐MN Reprogrammed the Psoriatic Lesion Transcriptome

To elucidate the transcriptional mechanisms underlying the therapeutic efficacy of **CPD3a**, transcriptomic RNA sequencing (RNA‐seq) was performed on dorsal skin lesions from IMQ‐induced psoriatic mice treated with H‐**CPD3a**‐MN and the untreated model group. The Volcano plot revealed 561 statistically significant differentially expressed genes (DEGs) in the H‐**CPD3a**‐MN group compared with the model group, with a symmetrical distribution of 278 upregulated and 283 downregulated transcripts (Figure 6A). The heatmap suggested that these DEGs produced distinct expression patterns that clearly segregated the treatment groups, confirming a robust transcriptional response to **CPD3a** (Figure 6B). Functional analysis linked these changes directly to the core pathology of psoriasis. Gene Ontology (GO) enrichment analysis showed that DEGs were enriched in keratinocyte differentiation, keratinization, inflammatory response, and immune system process (Figure 6C). The GO chord plot further linked differentially expressed mRNAs to specific biological processes, cellular components, and molecular functions (Figure 6D). For instance, *Lce3d*, a major component of the cornified envelope involved in the terminal stage of keratinization [45], was downregulated, indicating that H‐**CPD3a**‐MN effectively suppressed the hyperkeratosis in psoriatic mouse skin. Other immune‐related DEGs such as *Cxcl13* and *Ngp* were also significantly downregulated, reinforcing the anti‐inflammatory effect of **CPD3a**. Kyoto Encyclopedia of Genes and Genomes (KEGG) annotation and enrichment analysis highlighted significant suppression of critical inflammatory pathways, including IL‐17 signaling pathway, NF‐κB signaling pathway, and cAMP signaling pathway— all central to psoriasis pathogenesis (Figure 6E,F). In summary, the transcriptomic profile demonstrates that **CPD3a**‐MN reprogrammed the psoriatic lesion transcriptome by suppressing inflammatory networks and restoring normal epidermal differentiation.

**FIGURE 6:**
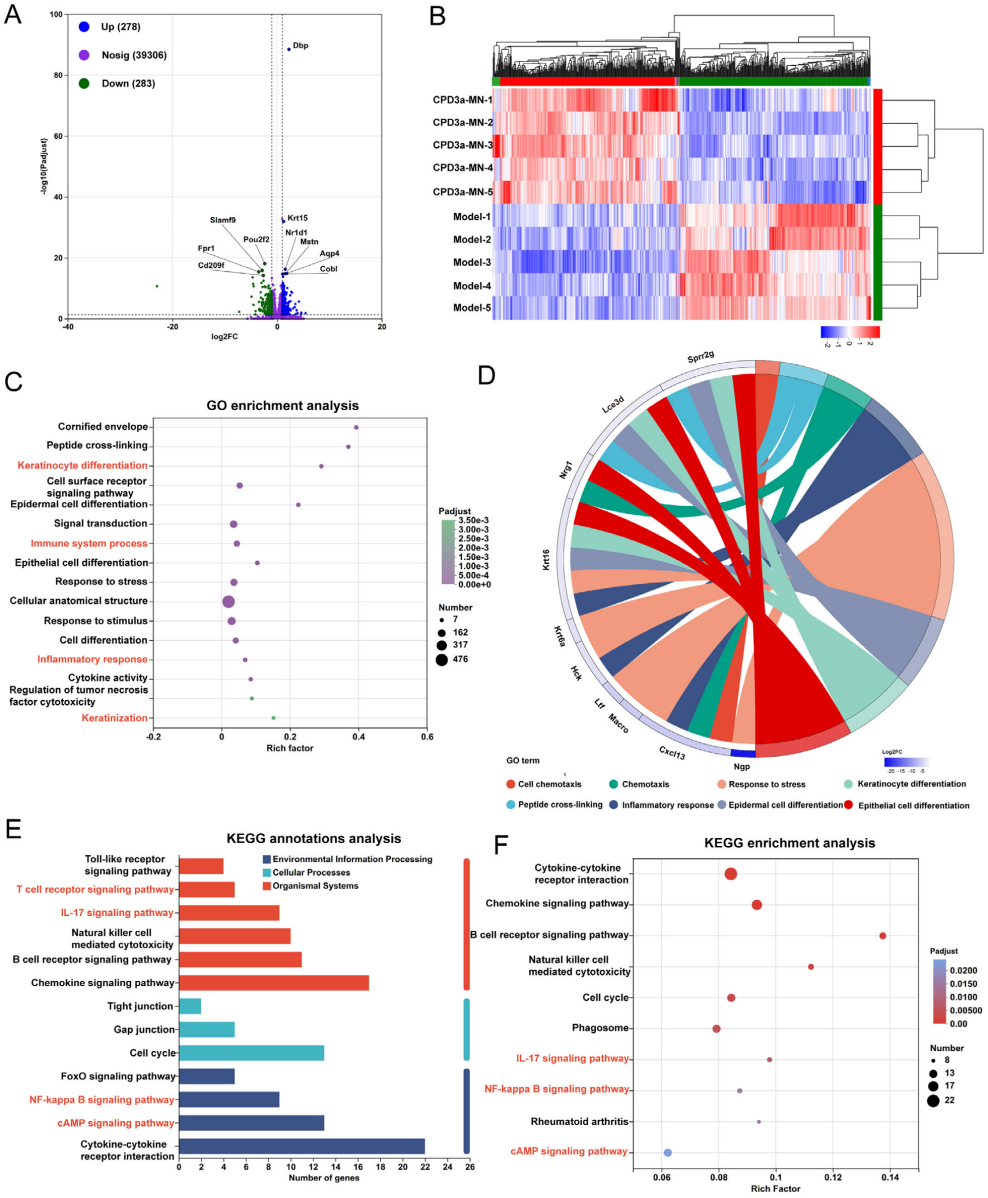
The transcriptomic analysis of dorsal skin lesions from psoriasis‐like mice by RNA‐seq. (A) The volcano plot and (B) the heatmap displaying differentially expressed genes (DEGs) between the H‐**CPD3a**‐MN and Model groups. (C) GO enrichment analysis of the DEGs. (D) GO Chord Diagram of the DEGs between the H‐**CPD3a**‐MN and Model groups. (E) KEGG annotation analysis of the DEGs between the H‐**CPD3a**‐MN and Model groups. (F) KEGG enrichment analysis of the DEGs. For each group, *n* = 5 independent samples.

### 2.8 CPD3a‐MN Treatment Shows Favorable Systemic Safety

To evaluate the potential systemic toxicity of the **CPD3a**‐MN treatment, a comprehensive safety assessment in the IMQ‐induced psoriasis mouse model was conducted, including histopathological examination, serum biochemistry, and hematological analysis. Histopathological analysis of major organs (spleen, lung, kidney, liver, and heart) via H&E staining seven days post‐treatment revealed no evidence of acute inflammation, necrosis, or significant structural damage in any treatment group compared to the healthy control (Figure 7A). The tissue morphology examination across all vital organs indicated an absence of treatment‐induced organo‐toxicity. Serum biochemical markers provided functional data complementary to the histopathological findings. Levels of aspartate aminotransferase (AST, Figure 7B), creatinine (CREA, Figure 7D), and blood urea nitrogen (BUN, Figure 7E) in the M‐**CPD3a**‐MN and H‐**CPD3a**‐MN groups remained within normal physiological ranges, suggesting preserved renal function and the absence of significant hepatocellular injury. Notably, a mild but observable elevation in alanine aminotransferase (ALT, Figure 7C) was detected in the H‐**CPD3a**‐MN group. However, this increase did not exceed pathological thresholds and was not accompanied by histological liver damage.

**FIGURE 7:**
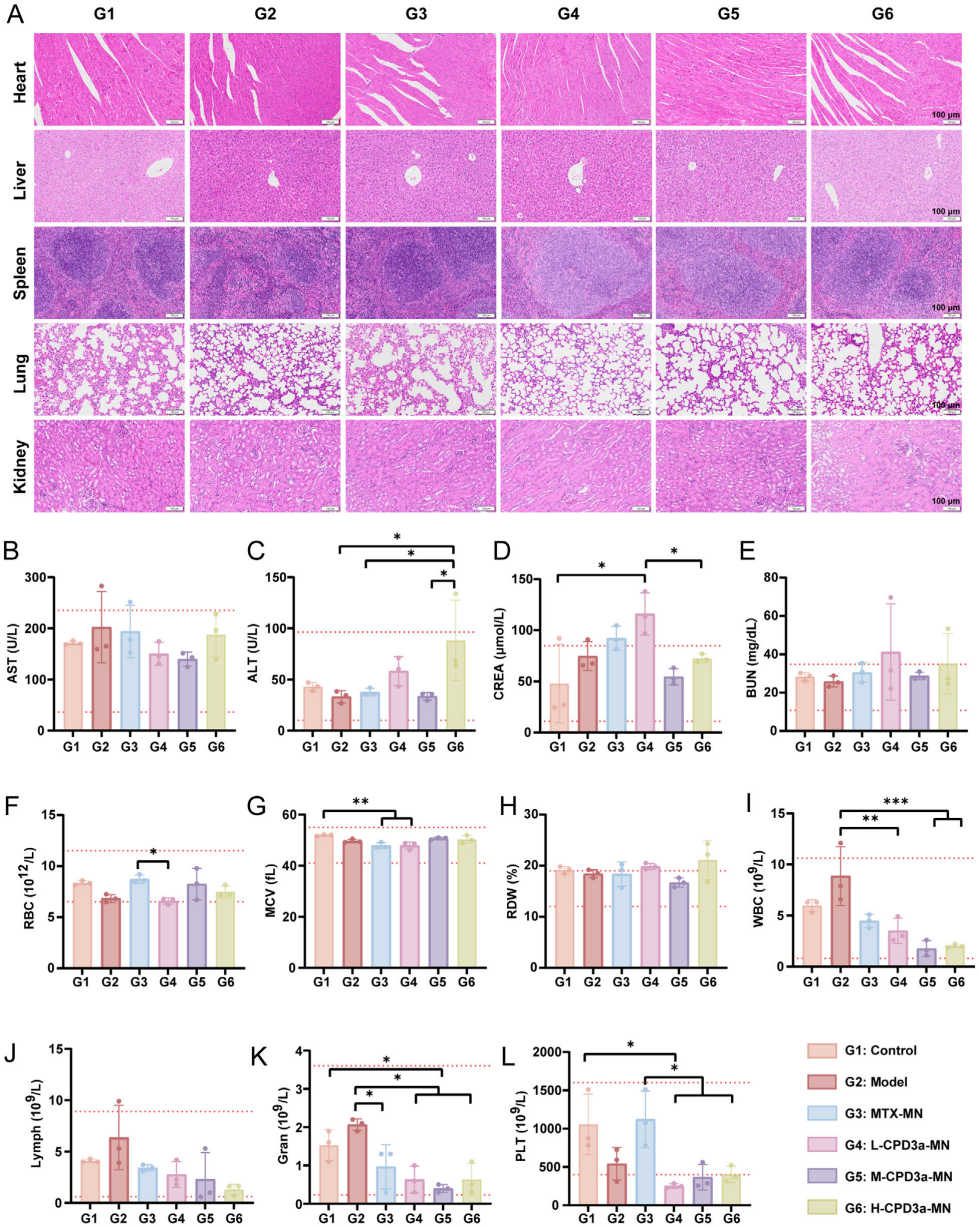
Systemic evaluation of **CPD3a**‐MN in IMQ‐induced psoriatic mice. (A) Representative H&E staining of major organs from each group (Scale bar: 100 µm). The serum biochemical indicators of each group, including (B) Aspartate aminotransferase (AST), (C) Alanine aminotransferase (ALT), (D) Creatinine (CREA), (E) Blood urea nitrogen (BUN). The hematological parameters of the mice in each group, including (F) Red blood cell (RBC), (G) Mean corpuscular volume (MCV), and (H) Red cell distribution width (RDW), (I) White blood cell (WBC), (J) Lymphocyte (Lymph), (K) Granulocyte (Gran), (L) Platelet (PLT). The red dash line indicates the normal reference range. Data are shown as the mean ± SD. For each group, *n* = 3 independent samples in (B–L). ^*^ *p* < 0.05, ^**^ *p* < 0.01, ^***^ *p* < 0.001.

Hematological analysis further delineated the systemic impact of treatment. Parameters, including red blood cell count (RBC, Figure 7F), mean corpuscular volume (MCV, Figure 7G), and red cell distribution width (RDW, Figure 7H), fell within standard reference ranges, indicating no adverse effects on erythropoiesis or erythrocyte integrity. However, a reduction was observed in several indicators in **CPD3a**‐MN‐treated groups compared to the control. Specifically, total white blood cell count (WBC, Figure 7I), lymphocyte count (Lymph, Figure 7J), granulocyte count (Gran, Figure 7K), and platelet count (PLT, Figure 7L) were lower in the **CPD3a**‐MN treatment groups. These reductions did not indicate profound cytopenia or immunosuppression. Collectively, these findings demonstrate that **CPD3a**‐MN treatment exhibits a favorable systemic safety profile, with no significant organ toxicity and only minor, clinically insignificant hematological alterations.

## 3 Conclusion

In this study, two classes of cordycepin derivatives distinguished by the glycosidic bond were designed and synthesized. Three compounds (**CPD2c, 2d, and 2e**) with the C─N glycosidic bond exhibited significantly enhanced anti‐inflammatory activities but showed comparable metabolic stability to cordycepin, indicating limited potential for further development. In contrast, a C‐nucleoside analog of cordycepin, **CPD3a**, was identified not only as highly potent in inhibiting pro‐inflammatory cytokine release, but remarkably metabolically stable in human and mouse liver microsomes. Moreover, **CPD3a** demonstrated an excellent PK profile in mice following IV or PO administration. A small amount of **CPD3a** deamination metabolite (**CPD3a‐M**) was detected in plasma only after oral administration, reflecting high resistance to in vivo ADA metabolism.

**CPD3a** was subsequently evaluated for the topical treatment of psoriasis through formulated microneedle patch. In the IMQ‐induced psoriatic mouse model, treatment with **CPD3a**‑MN elicited a robust, dose‑dependent therapeutic response, as evidenced by a significant reduction in clinical disease severity (PASI score), histological amelioration of epidermal hyperplasia and hyperkeratosis. Also, **CPD3a**‑MN caused normalization of systemic immune activation, indicated by the reversal of splenomegaly.

The efficacy of **CPD3a**‑MN arose from a coordinated intervention on multiple pathological pathways. It directly inhibited pathological keratinocyte proliferation. Furthermore, **CPD3a**‑MN exerted broad immunomodulatory effects by disrupting the IL‑17/NF‑κB inflammatory axis, downregulating key cytokines locally and systemically. It also rebalanced splenic leukocyte populations—suppressing pro‑inflammatory neutrophils and dendritic cells while expanding Tregs and M2‑phenotype macrophages. Additionally, **CPD3a**‑MN treatment reduced oxidative stress by decreasing epidermal ROS accumulation, which coincided with AMPK phosphorylation, upregulation of NRF2, implicating the AMPK/NRF2 antioxidant pathway in the observed antioxidant effect. Critically, the potent therapeutic activity of **CPD3a**‐MN was accompanied by a favorable safety profile with no significant histopathological damage in major organs.

In summary, **CPD3a‐**MN, as a promising therapeutic system, targets the complex pathophysiology of psoriasis through coordinated immunomodulation, oxidative stress reduction, and genomic reprogramming of inflamed skin while demonstrating favorable systemic safety. Collectively, these findings established a solid foundation for developing **CPD3a**‐based therapies against psoriasis and other immune‐mediated inflammatory diseases.

## 4 Experiments

### 4.1 Materials

All reagents were purchased from commercial suppliers and used without further purification. ^1^H and ^13^C NMR spectral data were recorded in DMSO‐*d6* with a Bruker 400 MHz spectrometer using tetramethylsilane (TMS) as the internal standard. All products were characterized by high‐resolution mass spectrometry (HRMS) recorded on an ABI/Sciex QStar mass spectrometer (AB SCIEX, Boston, United States of America(USA)). Polyvinylpyrrolidone (PVP, K‐30) was purchased from Beijing Solarbio Science & Technology Co., Ltd. Polyvinyl alcohol (PVA) and methotrexate (MTX) were obtained from Sigma–Aldrich Trading Co., Ltd. Imiquimod (IMQ) was acquired from Shanghai Titan Scientific Co., Ltd.

The human monocytic cell line THP‐1 (CVCL_0006) was obtained from Procell Life Science & Technology Co., Ltd. (Wuhan, China). Human immortalized epidermal cell line HaCaT (CVCL_0038) and national institutes of health 3T3 cell line NIH3T3 (CVCL_0594) were purchased from Shanghai Jiwei Biological Technology Co., Ltd. (Shanghai, China). Dulcecco's Modified Eagle Medium (DMEM) was obtained from Meilunbio (Dalian, China); RPMI‑1640 medium, fetal bovine serum (FBS), and penicillin–streptomycin (10,000 U/mL) were purchased from Gibco (Thermo Fisher Scientific, USA). NRF2 rabbit mAb, and phospho‐AMPK alpha 1/2 (Thr183/Thr172) rabbit pAb were obtained from Chengdu Zhengneng Biotechnology Co., Ltd. AMPK alpha 1 Rabbit mAb and GAPDH Rabbit mAb were purchased from Beyotime Biotechnology (Shanghai, China). Phorbol 12‑myristate 13‑acetate (PMA) was obtained from Yuanye Bio‐Technology Co., Ltd. (Shanghai, China). Lipopolysaccharides (LPS) were obtained from TargetMol (Shanghai, China). 4% paraformaldehyde was from Wuhan Servicebio Technology Co., Ltd. Erythrocyte lysis buffer was purchased from Absin Bioscience Inc (Shanghai). Mouse IL‐17A ELISA kit, mouse IL‐23 ELISA kit, mouse TNF‐α ELISA kit, and mouse IL‐6 ELISA kit were acquired from Jianglai Biotechnology Co., Ltd (Shanghai, China). Anti‐mouse CD16/32, FVD455UV, BV510 anti‐mouse CD45, Spark Blue 574 anti‐mouse CD3, BV570 anti‐mouse CD4, PE/Fire744 anti‐mouse CD11b, BV605 anti‐mouse CD11c, APC/Fire810 anti‐mouse F4/80, PE/Fire640 anti‐mouse Ly‐6G, PE anti‐mouse FoxP3, BV785 anti‐mouse CD206, fixation /permeabilization concentrate, fixation /permeabilization diluent, permeabilization buffer, and human TNF‐α and IL‐6 ELISA MAX Deluxe kits were acquired from Biolegend Co., Ltd. Cell Counting Kit 8 (CCK‐8) reagent was purchased from BGBioscience Co., Ltd. The bicinchoninic acid (BCA) Protein Assay Kit was purchased from Shanghai Yuanye Bio‐Technology Co., Ltd. (Shanghai, China). The BeyoWB 80‑min Electrophoresis, Transfer and Western Detection Kit was obtained from Beyotime Biotechnology.

### 4.2 Synthesis of Cordycepin Derivatives

The chemical structures of **22** cordycepin derivatives are shown in Figure 1A. The chemical syntheses are detailed in supporting information Schemes S1–S6. To ensure the accuracy and integrity of the characterizations, all final products were analyzed by NMR and HRMS. The corresponding ^1^H and ^1^ ^3^C NMR spectra are provided in Figures S8–S44.

### 4.3 Evaluation of Anti‐Inflammatory Activity In Vitro

THP‐1 cells were grown in RPMI‐1640 medium containing 10% fetal bovine serum, 1% penicillin–streptomycin, and 50 µm 2‐mercaptoethanol. Cells were seeded into 96 well plates at the density of 5 × 10^5^ cells/mL with medium (containing 500 nm PMA) and incubated at 37°C, 5% CO_2_ incubator for 24 h. The cell culture medium was replaced with fresh RPMI 1640 medium. Cells were first treated with the test compounds, followed by incubation at 37 °C under 5% CO_2_ for 1 h, and then treated with 1 µg/mL LPS at 37 °C for either 4 h (for TNF‑α analysis) or 12 h (for IL‑6 analysis). After that, the culture supernatant was collected and stored at −80°C until analysis. TNF‑α and IL‑6 levels were measured using ELISA MAX Deluxe kits according to the manufacturer's protocol. The inhibition rate (%) for each cytokine was calculated according to the following formula:

$$\begin{array}{ccc} \mathrm{Inhibition}\;\mathrm{rate}\;(\%) & = & 1-[(\mathrm{Conc}\;\mathrm{compound}-\mathrm{Conc}\;\mathrm{control})/\\ & & (\mathrm{Conc}\;\mathrm{LPS}-\mathrm{Conc}\;\mathrm{control})]\times 100\% \end{array}$$

where:

Conc _LPS_ is the cytokine concentration in the LPS‑stimulated group (without compound treatment),

Conc_compound_ is the cytokine concentration in the compound‑treated and LPS‑stimulated group,

Conc_control_ is the cytokine concentration in the unstimulated control group (without LPS and compound).

### 4.4 Cytotoxicity Measurements

THP‐1 cell viability was assessed using the CCK‐8 assay. Briefly, cells were seeded in 96‐well plates at a density of 1 × 10^4^ cells/mL and incubated for 24 h. Subsequently, the cells were treated with the indicated compounds or vehicle control for the indicated durations (48 or 72 h). Following treatment, 10 µL of the CCK‐8 reagent was added directly to each well, and the plates were incubated at 37°C for 4 h. The absorbance of the formazan dye produced was then measured at 450 nm by microplate reader. Cell viability was calculated as a percentage relative to the untreated control group, with the background absorbance from culture medium alone subtracted.

HaCaT and NIH3T3 cells were cultured in DMEM supplemented with 10% fetal bovine serum (FBS) and 1% penicillin–streptomycin at 37°C in a 5% CO_2_ incubator. Cell viability was assessed using the CCK‑8 assay. Cells were seeded in 96‑well plates at a density of 5 × 10^4^ cells/mL and incubated for 24 h to allow cell attachment. The cells were then treated with various concentrations of **CPD3a** or **CPD3a**+PVP (**CPD3a** concentration: 1.56, 3.13, 6.25, 12.5, 25, 50, 100, and 200 µm) or PVP (with the same dilution ratios as those used for the **CPD3a**+PVP groups) for 24 h. After treatment, the culture medium was replaced with fresh DMEM containing 10% CCK‑8 reagent and incubated at 37°C for 2 h. The absorbance of the formazan dye was measured at 450 nm using a microplate reader. Cell viability was calculated as a percentage relative to the untreated control group, with the background absorbance from culture medium alone subtracted.

### 4.5 Metabolic Stability Measurements

The metabolic stability of the test compound was assessed in human and mouse liver microsomes. Briefly, the compound (1 µm) was incubated with liver microsomes (0.5 mg/mL) in the presence of an NADPH‐regenerating system at 37°C. Aliquots were collected at predetermined time points over 60 min, and the reactions were quenched with cold acetonitrile. The remaining parent compound at each time point was quantified using liquid chromatography‐tandem mass spectrometry (LC‐MS/MS). The in vitro half‐life (T_1/2_) and intrinsic clearance (CL_int_) were then calculated from the natural logarithm of the percentage of compound remaining plotted against time.

### 4.6 PK and Tissue Distribution Studies In Mice

Male C57BL/6 mice were purchased from Shanghai Shengchang Biotechnology Co., Ltd. The test compound, formulated in a vehicle of DMSO/EtOH/PEG300/0.9% NaCl (5/5/40/50, v/v/v/v), was administered to mice (*n* = 3) at a single intravenous (IV) dose of 5 mg/kg or oral (PO) dose of 25 mg/kg. After IV administration, blood samples (∼30 µL) were collected via the jugular or submandibular vein at 0.083, 0.25, 0.5, 1, 2, 4, 6, 8, and 24 h. For the PO group, blood was collected at 0.25, 0.5, 1, 2, 4, 6, 8, and 24 h. All samples were placed into EDTA‐K2 tubes, centrifuged at 14 000 rpm for 5 min to obtain the plasma, which was stored at −70°C until bioanalysis. The concentration of the test compound or the metabolite in plasma was quantified by LC‐MS/MS. The PK parameters were calculated by non‐compartmental analysis using Phoenix WinNonlin. Oral bioavailability (F) was determined from the dose‐normalized AUC_0‐∞_ ratio following PO and IV administration.

The tissue distribution of **CPD3a** was investigated in male C57BL/6 mice (*n* = 3 per time point) following a single oral dose of 15 mg/kg. At predetermined time points post‐dosing (0.25, 1, and 6 h), animals were euthanized, and tissues—including plasma, liver, small intestine, stomach, pancreas, colon, kidney, lung, and brain—were immediately collected. Each tissue sample was homogenized in an ice‐cold buffered saline solution, and the resulting homogenates were stored at −80°C until analysis. The concentrations of the compound in tissue homogenates and plasma were determined using a validated LC‐MS/MS method.

### 4.7 Preparation of Microneedle Patches

**CPD3a**‐loaded microneedles (**CPD3a**‐MN) were fabricated by solvent casting and vacuum‐assisted micro‐molding technique. A polydimethylsiloxane (PDMS) mold featuring an array of conical cavities (needle height: ∼600 µm, base width: ∼300 µm, tip‐to‐tip spacing: ∼550 µm) was used as the negative template. Briefly, the composite hydrogel mixture (200 µL) comprising PVP (10% w/v) and the active compound **CPD3a** was precisely dispensed into the mold cavities. Then the mold was put into a 50 mL centrifuge tube and was centrifuged at 3500 rpm for 5 min. Vacuum was applied to ensure complete filling and removal of air bubbles by a vacuum drying chamber (DZF‐6050, Shanghai YIHENG Technical Co., Ltd). Subsequently, a backing layer solution of a mixture of 20% PVA (w/v) and 10% PVP was cast over the filled mold. The assembled system was dried in 0.1 kPa vacuum for 12 h at 37°C. After complete drying, the microneedle patch was carefully demolded, yielding a flexible array with drug‐loaded needle tips. The methotrexate loaded microneedle (MTX‐MN) and FITC‐labeled **CPD3a**‐MN (FITC‐**CPD3a**‐MN) were prepared in a manner consistent with the **CPD3a**‐MN preparation described above.

### 4.8 Characterization of Microneedle Patches

The surface morphology of **CPD3a**‐MN was observed through scanning electron microscopy (SEM, GeminiSEM 360, Zeiss, Germany) at 5 KV. A texture analyzer (Shanghai TengBa Instrument Technology Co., LTD) was utilized to analyze the mechanical strength of **CPD3a**‐MN. To visualize the spatial distribution of the drug within the needles, the MN patch, fabricated from a solution containing rhodamine‐labeled PVP and fluorescein isothiocyanate isomer (FITC)‐labeled PVA/PVP, was imaged by confocal laser scanning microscopy (CLSM, Stellaris 5, Leica, Germany). To verify the spatial distribution of the drug in the microneedles, FITC‐labeled **CPD3a** was used as a model drug, and its distribution in the MN tip was observed using the CLSM. The insertion capability was validated by murine skin in vivo. After pressing **CPD3a**‐MN to the mouse skin for 20 s, the microneedle was removed, and the skin condition was recorded using an electronic device at various time points (0, 2, 5, 10, 15, and 25 min). Application of the patch, followed by histological analysis (hematoxylin and eosin staining), confirmed the creation of microchannels. To investigate the skin penetration of MN, the FITC‐**CPD3a**‐MN were inserted into fresh mouse skin and firmly pressed for 20 s. Using the skin tissue plane at the site of FITC‐**CPD3a**‐MN insertion as the reference, green fluorescence signals were observed at various depths (at 20 µm intervals) by the CLSM. The concentration of **CPD3a** in the needle tips and baseplate was detected by high‐performance liquid chromatography (HPLC, Alliance e2695, Waters, USA) at a UV absorption wavelength of 244 nm. To determine the content of **CPD3a** in the skin, **CPD3a**‐MN was applied to fresh mouse skin with firm pressure for 20 s. After 1 h, the treated skin was collected, homogenized in 0.5 mL of acetonitrile, and centrifuged at 12 000 rpm for 20 min. The resulting supernatant was analyzed by HPLC to quantify **CPD3a** content. To evaluate the in vitro release profile of **CPD3a** from the dissolving microneedles, **CPD3a**‐MN were placed in 30 mL PBS at 37°C under 100 rpm (*n* = 3), and samples were taken and detected at different time points (0, 0.5, 1, 2, 4, 6, 8, 10, 12, 24, and 48 h). The in vitro transdermal permeation of **CPD3a**‐MN was investigated using the Franz diffusion cell method. Prior to the experiment, 15 mL of PBS at 32°C was added to the receptor compartment. Mouse skin was cut to an appropriate size and mounted between the donor and receptor compartments with the stratum corneum facing upward. 500 µL solution was collected at 0.25, 0.5, 1, 2, 4, and 6 h under conditions of 350 rpm and 32°C. Subsequently, 500 µL fresh phosphate‐buffered saline (PBS) was immediately replenished into the receptor compartment. Samples at each time point were centrifuged at 12 000 rpm for 10 min, and the supernatant was then analyzed by HPLC to determine the **CPD3a** concentration. The morphology of **CPD3a**‐MN degradation at different time points (0, 1, 10, 20, and 30 min) in vivo was observed by SEM.

### 4.9 Animal Model Establishment

Female BALB/c mice (6–8 weeks old) were purchased from Zhejiang Vital River Laboratory Animal Technology Co., Ltd. A psoriasis‐like mouse model was induced on the shaved dorsal skin by daily topical application of 5% IMQ cream (62.5 mg) for seven consecutive days.

All animal procedures were conducted in accordance with ethical regulations and guidelines of Lingang Laboratory and were approved by the Institutional Animal Care and Use Committee (IACUC) of Lingang Laboratory (Protocol No.: LGLSP‐2023–010).

### 4.10 In Vivo Pharmacodynamics and Biocompatibility

Female BALB/c mice were randomly assigned to six experimental groups (*n* = 5 per group): a naïve control group (G1), an IMQ‐induced model group (G2), a positive control group receiving methotrexate‐loaded microneedles (G3: MTX‐MN, 5 mg/kg), and three intervention groups treated with **CPD3a**‐MN at low (G4: L‐**CPD3a**‐MN, 2 mg/kg), medium (G5: M‐**CPD3a**‐MN, 5 mg/kg), and high doses (G6: H‐**CPD3a**‐MN, 10 mg/kg). Psoriasis‐like mouse model was induced for seven consecutive days. Microneedle patches were administered intradermally to the treatment area every day, starting on day 1 of IMQ induction.

Disease severity was evaluated daily according to the Psoriasis Area and Severity Index (PASI), scoring three parameters: erythema, keratinization, and desquamation. Body weight was monitored every day throughout the study. On day 7, mice were euthanized. Blood was collected, and serum was isolated for subsequent cytokine (IL‐17A, IL‐23, TNF‐α, and IL‐6) and biochemical analyses.

### 4.11 Assessment of Systemic Immune and Inflammatory Responses

Dorsal skin lesions were collected, fixed in formalin for hematoxylin and eosin (H&E) staining, and immunohistochemistry. The residual skin tissues were snap‐frozen for transcriptomic analysis.

At the end of in vivo pharmacodynamics evaluation, all the spleens from mice were harvested, weighed, and processed for flow cytometric immunophenotyping. For the analysis of immune cell subsets, after spleens (100 mg) were ground into the single cell suspension by a single cell suspension preparation instrument (Shanghai Jingxin Industrial Development Co., Ltd), cells were collected and mixed with erythrocyte lysis buffer to exclude the effect of erythrocytes. Subsequently, single cell suspension was counted and blocked by anti‐mouse CD16/32 antibody for 30 min. After washed by DPBS buffer, cells were stained with cell surface flow antibodies (FVD455UV, BV510 anti‐mouse CD45, Spark Blue 574 anti‐mouse CD3, BV570 anti‐mouse CD4, PE/Fire744 anti‐mouse CD11b, BV605 anti‐mouse CD11c, APC/Fire810 anti‐mouse F4/80, and PE/Fire640 anti‐mouse Ly‐6G) for 30 min on ice in the dark. Then, cells were fixed by fixation /permeabilization concentrate diluted by fixation /permeabilization diluent for 40 min and mixed with permeabilization buffer for 30 min on ice. Intracellular cytokines and transcription factors were then stained with PE anti‐mouse FoxP3 and BV785 anti‐mouse CD206 for 30 min on ice in the dark. Finally, the stained cells were washed and analyzed using a spectral flow cytometer (Aurora, Cytek Biosciences, USA). In addition, the inflammatory factors (IL‐17A, IL‐23, TNF‐α, and IL‐6) in spleen tissues were also analyzed according to the ELISA kits' instructions.

### 4.12 Cellular Thermal Shift Assay

The HaCaT cells were divided into two groups: a control group treated with PBS and a **CPD3a** group treated with 20 µm **CPD3a** in PBS. After 12 h of incubation, the harvested cells were gently rinsed in a pre‐chilled PBS solution that contained a protease inhibitor and subsequently divided into five aliquots. The cell suspensions were equally distributed into five 1.5 mL centrifuge tubes and subjected to different temperature conditions (45°C, 50°C, 55°C, 60°C, and 65°C). Following 3 min of heat treatment, the samples were immediately placed in liquid nitrogen. Proteins were isolated and then detected by Western blotting. The entire experiment was repeated three times independently to ensure reproducibility.

Total proteins from HaCaT cells were quantified using the BCA method. The proteins were then subjected to sodium dodecylsulfate‐polyacrylamide gel electrophoresis (SDS‐PAGE), transferred to polyvinylidene difluoride (PVDF) membranes, and blocked. Following this, the membranes were incubated with primary antibodies AMPK and GADPH and secondary antibodies. The proteins were detected using chemiluminescence, and protein expression was analyzed. Band densities were quantified by Image J (Version:1.54f). The internal control protein GADPH's densitometric value was used to standardize the protein level.

### 4.13 Statistical Analysis

Statistical analyses were performed via GraphPad Prism 8. Data were presented as the mean ± standard deviation (SD). C*t*omparisons across multiple groups were assessed by one‐way analysis of variance (ANOVA). A p‐value of less than 0.05 was considered statistically significant, with specific thresholds denoted as follows: ^*^ *p* < 0.05, ^**^ *p* < 0.01, and ^***^ *p* < 0.001.

## Author Contributions

**Xujie Sun**: investigation, formal analysis. **Tianqun Lang**: conceptualization, supervision, funding acquisition, project administration, resources, writing – review and editing. **Zongyan He**: investigation, formal analysis. **Xinyue Shao**: conceptualization, methodology, data curation, investigation, validation, formal analysis, visualization, writing – review and editing, writing – original draft. **Yuanchen Zhong**: investigation, formal analysis. **Yuanchao Xie**: conceptualization, funding acquisition, project administration, resources, writing – review and editing, supervision. **Lixuan Yin**: investigation, formal analysis. **Wenfang Pan**: conceptualization, methodology, data curation, investigation, validation, formal analysis, visualization, writing – original draft, writing – review and editing.

## Conflicts of Interest

The authors declare no conflicts of interest.

## Supporting information




**Supporting File**: advs77012‐sup‐0001‐SuppMat.docx.

## Data Availability

The data that support the findings of this study are available from the corresponding author upon reasonable request.
