# Supplementary material for: A C‐Nucleoside Analogue of Cordycepin With High Metabolic Stability and Potent Anti‐Psoriatic Activity via Microneedle Delivery
Source: Adv Sci (Weinh). 2026 Aug 5;13(60):e77012. doi: 10.1002/advs.77012 (PMC13440205; doi:10.1002/advs.77012)
Supplement: Supplementary file 1 [file ADVS-13-e77012-s001.docx]

Supporting Information

A C-nucleoside Analogue of Cordycepin with High Metabolic Stability and Potent Anti-Psoriatic Activity via Microneedle Delivery

*Wenfang Pan ^1,2,#^, Xinyue Shao ^1,3,#^, Yuanchen Zhong ^1,2^, Xujie Sun ^1^, Lixuan Yin ^1,3^, Zongyan He^1,3^, Tianqun Lang ^1,*^, Yuanchao Xie ^1,*^*

1. Lingang Laboratory, Shanghai 200031, China;

2.School of Physical Science and Technology, ShanghaiTech University, Shanghai 201210, China

3. School of Life Science and Technology, ShanghaiTech University, Shanghai 201210, China

*. Correspondances: Tianqun Lang ([langtq@lglab.ac.cn](mailto:langtq@lglab.ac.cn)),

Yuanchao Xie ([xieyuanchao@lglab.ac.cn](mailto:xieyuanchao@lglab.ac.cn))

#. These authors contributed equally to this work.

**Content**

# 1. Supporting figures and tables

## Table S1. TNF-α inhibition rates of cordycepin derivatives^a^

| **Compounds** | **TNF-α inhibition (%)^b^** | | **Cell viability (%)^c^** |
| --- | --- | --- | --- |
|  | **25 μM** | **2.5 μM** | **25 μM** |
| **CPD1a** | 16.7±1.2 | 13.0±7.8 | 104.9±12.1 |
| **CPD1b** | ＜5 | ＜5 | 111.4±8.9 |
| **CPD1c** | 17.6±3.4 | ＜5 | 105.3±5.2 |
| **CPD1d** | 5.9±6.8 | ＜5 | 107.1±0.7 |
| **CPD1e** | 9.2±2.9 | ＜5 | 111.5±4.3 |
| **CPD1f** | ＜5 | ＜5 | 114.7±6.8 |
| **CPD1g** | ＜5 | ＜5 | 101.6±5.1 |
| **CPD1h** | 7.4±4.6 | ＜5 | 103.2±3.7 |
| **CPD1i** | 14.2±3.3 | 7.2±1.5 | 106.1±5.1 |
| **CPD1j** | 8.2±5.1 | 9.4±6.9 | 103.7±2.7 |
| **CPD2a** | 39.4±7.7 | 8.9±5.8 | 93.4±10.6 |
| **CPD2b** | 14.6±5.4 | 17.7±8.8 | 104.1±12.3 |
| **CPD2c** | 80.0±4.3 | 31.3±8.4 | 104.9±9.0 |
| **CPD2d** | 75.3±1.7 | 24.8±4.0 | 77.5±10.2 |
| **CPD2e** | 74.4±5.4 | 33.9±2.3 | 117.3±7.5 |
| **CPD3a** | 84.3±5.2 | 24.7±8.1 | 100.8±8.5 |
| **CPD3b** | 13.7±4.6 | 10.5±6.8 | 94.7±11.2 |
| **CPD3c** | 13.0±3.9 | 14.8±7.3 | 105.3±4.6 |
| **CPD3d** | 16.6±5.2 | 21.3±9.0 | 103.7±6.8 |
| **CPD3e** | 14.3±4.1 | 7.7±2.3 | 97.7±2.3 |
| **CPD3f** | 11.3±3.5 | 11.5±5.7 | 99.5±15.5 |
| **CPD3g** | 8.3±2.9 | ＜5 | 98.9±12.4 |
| **Cordycepin** | 31.0±0.6 | ＜5 | 100.1±3.6 |
| **Dexamethasone^d^** | 81.9±4.0 | | 102.5±7.9 |

^a^ Data were shown as the mean±SD. For each group, n = 3 independent samples.;

^b^ means TNF-α cytokine release experiment was conducted by Elisa assay;

^c^ means cell viability was assayed by CellTiter-Glo;

^d^ means dexamethasone group was tested in 10 μM.

## Table S2. TNF-α EC_50_ and cytotoxicity of the selected compound in THP-1 cell ^a^

| **Compounds** | **TNF-α IC_50_ (μM)** | **IL-6 IC_50_ (μM)** | **CC_50_ (μM)** |
| --- | --- | --- | --- |
| **CPD2c** | 4.73 ± 1.41 | 22.57± 3.50 | 60.3±5.7 |
| **CPD3a** | 3.37 ± 1.07 | 6.74±1.12 | 102.6±10.3 |
| **Cordycepin** | ＞25 | ＞50 | 127.4±11.0 |

^a^ Data were shown as the mean±SD. For each group, n = 3 independent samples;

## Table S3. Microsomal stability of CPD2c, CPD3a and Cordycepin ^a^

| **Compounds** | **Species** | **T_1/2_ (min)** | **CL_int(liver)_ (mL/min/kg)** | **E_h_%** |
| --- | --- | --- | --- | --- |
| **CPD2c** | Human | 52.3 | 33.2 | 61.6 |
|  | Mouse | 18.2 | 299.6 | 76.9 |
| **CPD3a** | Human | 1145.6 | 1.5 | 6.8 |
|  | Mouse | 1059.8 | 5.2 | 5.4 |
| **Cordycepin** | Human | 27.2 | 63.9 | 75.5 |
|  | mouse | 15.5 | 351.3 | 79.6 |

^a^ Data were shown as the mean value. For each group, n = 2 independent samples.

## Table S4. In vivo pharmacokinetic parameters of CPD3a ^a^

| **Analyte** | **Group** | **T_max_ (h)** | **T_1/2_ (h)** | **MRT_0-∞_(h)** | **C_max_(ng/mL)** | **AUC_0-t_ (h*ng/mL)** | **AUC_0-∞_ (h*ng/mL)** | **CL (mL/min/kg)** | **Vss (L/kg)** | **F%** |
| --- | --- | --- | --- | --- | --- | --- | --- | --- | --- | --- |
| **CPD3a** | IV  5 mpk |  | 11.5 | 11.5 | 1810 | 2128 | 2546 | 32.7 | 22.5 |  |
|  | PO  25mpk | 0.5 | 8.9 | 9.0 | 1647 | 8705 | 9754 |  |  | 81.8 |
| **CPD3a-M** | IV  5 mpk |  |  |  | 4.5 |  |  |  |  |  |
|  | PO  25mpk | 2 | 3.7 | 4.6 | 111 | 470 | 501 |  |  |  |

^a^ Data were shown as the mean value. For each group, n = 3 independent samples.

## Table S5. In vivo pharmacokinetic parameters of Cordycepin ^a^

| **Analyte** | **Group** | **T_max_ (h)** | **T_1/2_ (h)** | **MRT_0-∞_ (h)** | **C_max_**  **(ng/mL)** | **AUC_0-t_ (h*ng/mL)** | **AUC_0-∞_ (h*ng/mL)** | **CL**  **(mL/min/kg)** |
| --- | --- | --- | --- | --- | --- | --- | --- | --- |
| **Cordycepin** | IV 5 mpk |  |  |  | BLQ^b^ |  |  |  |
|  | PO 25 mpk |  |  |  | BLQ^b^ |  |  |  |
| **3′-deoxyinosine** | IV 5 mpk |  | 1.9 | 1.5 | 11287 | 3665 | 3795 | 22.5 |
|  | PO 25 mpk | 0.3 | 1.6 | 2.1 | 4657 | 4549 | 4625 |  |

^a^ Data were shown as the mean value. For each group, n = 3 independent samples.

^b^ BLQ means Below the Limit of Quantification

## Table S6. Observations of skin irritation and skin corrosion on mice skin.

| Patch | Number of samples | Skin corrosion (SCT) | Skin irritation (SIT) |
| --- | --- | --- | --- |
| CPD3a-MN | 3 | No observation | No observation |

## Figure S1. The CLSM image of microneedle tips loaded FITC-labeled CPD3a.

## Figure S2. HPLC of CPD3a.

(A). UV-Vis full wavelength scan of **CPD3a**. The compound showed a characteristic absorption peak at 244 nm; (B). Linear regression analysis of concentration versus absorbance for **CPD3a**, used for standard curve construction. (C). HPLC chromatogram of **CPD3a** sample, showing the retention peak of the compound.

## Figure S3. Time-dependent images showing the recovery of skin pores at various time points after CPD3a-MN treatment.

## Figure S4. The CLSM image of FITC-CPD3a-MN at various skin penetration depths following insertion into mouse skin (Scale bar: 100 μm).

## Figure S5. The cumulative permeation amount and cumulative permeation percentage of CPD3a-MN. n = 3 independent samples in (A-B).

## Figure S6. Cytotoxicity of CPD3a, PVP, and CPD3a+PVP in HaCaT and NIH3T3. Data are presented as mean ± SD (n=3).

## Figure S7. CETSA analysis of AMPK binding by CPD3a in HaCaT cells.

(A) Representative Western blot images of soluble AMPK in HaCaT cells treated with PBS (Control) or **CPD3a** (20 μM) and heated at temperatures ranging from 45°C to 65°C. GAPDH served as the loading control. (B) Quantification of AMPK protein levels from three independent experiments. Data represent mean ± SD (n = 3). (C) Unprocessed Western blot membranes corresponding to the data shown in (A).

# 2. Chemistry

**Scheme S1.** The synthetic routes of compounds **CPD1f**~ **CPD1j**. Reagents and conditions: (a). TBSCl, imidazole, DMF; (b) Di-tert-butyl decarbonate, DMAP, TEA, DCM; (c) 3HF·TEA, THF, 50 ℃; (d) i. EDCI, pyridinium trifluoroacetate, DMSO; ii. 37% HCHO in H_2_O, NaOH, 1,4-dioxane and H_2_O (*v: v*=10:1); iii. NaBH_4_, MeOH; (e) TBDPSCl, imidazole, DMF; (f) DMTrCl, TEA, DCM; (g) 1.0 M TBAF in THF, THF; (h) i. Tf_2_O, pyridine, DCM, -20 ℃; ii. Tetrabutylammonium borohydride, toluene, 85 ℃, for **8a**; (i) i. Tf_2_O, pyridine, DCM, -20 ℃; ii. 1M TBAF solution in THF, THF, for **8b**; (j) i. Tf_2_O, pyridine, DCM, -20 ℃; ii. LiCl, DMF, 40 ℃, for **8c**; (k) i. EDCI, pyridine trifluoroacetate, DMSO; ii. Hydroxylamine hydrochloride, pyridine; iii. CDI, acetonitrile, 50 ℃, for **8d**; (l) Trifluoroacetic acid and dichloromethane (*v: v*=1:4).

The synthesis of cordycepin analogues **CPD1**, **2** and **3** series is depicted in **Scheme S1**~**S6**. Compounds **CPD1a**~**1e**, modified at the 3′-position, were prepared according to published procedures^1-5^. The route to 4'-substituted derivatives **CPD1f**~**1j** is outlined in **Scheme S1**. Starting from cordycepin, the 5′-OH group was selectively protected with TBSCl to furnish intermediate **1**, which was subsequently Boc‑protected to yield compound **2**. Removal of the TBS protecting group provided **3**. The 5′-hydroxyl in **3** was then subjected to Moffat oxidation, and the resulting aldehyde underwent an aldol reaction with formaldehyde, followed by reduction to afford the diol intermediate **4**^6^. Deprotection of intermediate **4** provided the 4′-hydroxylmethyl derivative **CPD1f**. To access analogues **CPD1g**~**1j**, a common intermediate **7** was first prepared from diol **4**. The less‑hindered 5′‑OH was selectively protected as its TBDPS ether, and the remaining primary hydroxyl was masked with a DMT group^6^. Cleavage of the TBDPS protecting group then furnished intermediate **7**. For compounds **CPD1g**~**1i,** the free hydroxyl of **7** was converted to a triflate ester. This intermediate was then subjected to reduction, fluorination, or chlorination, yielding the 4′-methyl (**8a**), 4′-fluoromethyl (**8b**), and 4′-chloromethyl (**8c**) derivatives, respectively^6^. Global deprotection of **8a**~**c** provided final compounds **CPD1g**~**1i**. The 4′-cyano derivative **CPD1j** was accessed via an alternative route. Swern oxidation of **7** gave the corresponding aldehyde, which was condensed with hydroxylamine to form the oxime. Dehydration with 1,1′‑carbonyldiimidazole furnished nitrile **8d**^7^, and final deprotection with TFA yielded **CPD1j**.

**Scheme S2.** The synthetic route of compounds **CPD2a**~**CPD2e**. Reagents and conditions: (a). 2, 6-Dichloropurine, BSA, TMSOTf, acetonitrile; (b). Benzylamine, TEA, 1, 4-dioxane, 60 ℃; (c) 7 N NH_3_/MeOH, 60 ℃.

The synthesis of C2-chlorine, C6-benzylamine derivatives **CPD2a~CPD2e** is illustrated in **Scheme S2**. Vorbrüggen glycosylation of 2, 6-dichloropurine with ribose intermediates **9a** or **9b** afforded the ester-protected nucleosides **10a** and **10b**, respectively. The C6‑chloro substituents in **10a** and **10b** were displaced via nucleophilic aromatic substitution (SN_Ar_) with variously substituted benzylamines under basic conditions, yielding intermediates **11a**~**11e.** Final deprotection with methanolic ammonia afforded the target compounds **CPD2a**~**CPD2e**.

**Scheme S3.** The synthetic route of compounds **CPD3a** and **CPD3b**. Reagents and conditions: (a). i. 2-Acetoxyisobutyryl bromide, acetonitrile and H_2_O (*v: v*=500:1); ii. NaOMe, MeOH; (b) 1.0 M Lithium triethylborohydride in THF, THF.

The C‑nucleoside analogues **CPD3a** and **CPD3b** were prepared as outlined in **Scheme S3**. Nucleosides **12a** and **12b** were prepared according to the methods described by Kim et al and Serge Van Calenbergh et al^8,9^. These nucleosides were subsequently converted to their epoxides **13a** and **13b**^10^, which upon reductive ring opening with lithium triethylborohydride yielded the title compounds **CPD3a** and **CPD3b**, respectively.

**Scheme S4.** The synthetic route of compound **CPD3c**. Reagents and conditions: (a). i. Tf_2_O, pyridine, DCM, -20 ℃; ii. Tetrabutylammonium borohydride, toluene, 85 ℃; (b). Conc. H_2_SO_4_, MeOH; (c) BnBr, 60% NaH, THF; (d) 80% AcOH in H_2_O, 80 ℃; (e) Ac_2_O, DMSO; (f) i. 7-Iodopyrrolo[2,1-f][1,2,4]triazin-4-amine, TMSCl, 2,0 M PhMgCl in THF, 0 ℃~-15 ℃; ii. 2.0 M i-PrMgCl in THF, RT~-15 ℃; (g) TESH, 46.5% BF_3_-Et_2_O, DCM, 0 ℃; (h) 10% Pd/C, AcOH.

The synthesis of 2′-*β*-methyl derivative **CPD3c** was accomplished via a multi-step procedure detailed in **Scheme S4**. In our initial attempt, we were not able to perform the 3′-deoxygenation under the same reaction conditions mentioned in **Scheme S3**. Consequently, we adopted a novel route to construct the 2′-*β*-methyl-3′-deoxyl ribose moiety **20**, followed by its coupling to the heterocyclic base. The starting material 2-*C*-methyl-*D*-ribono-1,4-lactone **14** was firstly converted to intermediate **15** using the reported method^11^, which was subsequently trifluoromethanesulfonylated and reduced with tetrabutylammonium borohydride to yield the 3′-deoxy intermediate **16**. Treatment of **16** with H₂SO₄ in methanol provided the 1′-O-methyl derivative **17**. A sequence of protection, deprotection, and oxidation steps then transformed **17** into the key ribonolactone intermediate **20**. For the coupling step, 7‑iodopyrrolo[2,1‑*f*][1,2,4] triazin‑4‑amine was firstly treated with PhMgCl and TMSCl to transiently protect the C6‑NH_2_ group. Subsequent halogen‑metal exchange with i-PrMgCl, followed by addition with lactone **20**, afforded the intermediate **21**. This intermediate was reduced with triethylsilane and boron trifluoride etherate to furnish **22** as the exclusive product. Finally, removal of the benzyl protecting groups delivered the target compound **CPD3c**.

**Scheme S5.** The synthetic route of compounds **CPD3d**. Reagents and conditions: (a). i. Conc. H_2_SO_4_, MeOH, 60 ℃; ii. K_2_CO_3_, MeOH; iii. BnBr, 60% NaH, THF; (b). 80% AcOH in H_2_O, 100°C; (c). Ac_2_O, DMSO; (d) i. 7-Iodopyrrolo[2,1-f][1,2,4]triazin-4-amine, TMSCl, 2,0 M PhMgCl in THF, 0 ℃~-15 ℃; ii. 2.0 M i-PrMgCl in THF, RT~-15 ℃; (e)2.0 M AlMe_3_ in toluene, 46.5% BF_3_-Et_2_O, DCM, 0 ℃; (f) 10% Pd/C, AcOH.

The preparation of compound **CPD3d** was started from intermediate **23** as depicted in **Scheme S5**. Sequential methanolysis, deprotection, and protection steps transformed **23** into the key intermediate **24**. Removal of the methyl group afforded **25**, which was subsequently oxidized to yield lactone **26**. Intermediate **27** was prepared from **26** using the same method described for **21**. Methylation of **27** with trimethylaluminum furnished the 1′‑methyl derivative **28**. Final deprotection provided the target compound **CPD3d**, obtained as an inseparable mixture.

**Scheme S6.** The synthetic route of compounds **CPD3e~CPD3h**. Reagents and conditions: (a). 1.6 M n-BuLi in hexanes, THF, -78 °C; (b). TESH, 46.5% BF_3_-Et_2_O, DCM, 0 ℃. (c). i. 7N NH_3_ in MeOH, 80 °C, for **CPD3e**; ii. 2-Chlorobenzylamine, TEA, ethanol, 50 °C, for **CPD3f**; (d). 90% Trifluoroacetic acid in H_2_O; (e). i. 2-Acetoxyisobutyryl bromide, acetonitrile and H_2_O (*v: v*=500:1); ii. Amberlite IRA 400 ion exchange resin, NaOH, H_2_O: THF (*v: v*=1:4); iii. 1.0 M Lithium triethylborohydride in THF, THF.

2-Chloro derivatives **CPD3e~3g** was synthesized as descripted in **Scheme S6**. Coupling of 7‑bromo‑2,4‑dichloropyrrolo [2,1‑*f*][1,2,4]triazine **29** with lactone **30** afforded intermediate **31**, which was subsequently reduced to give **32**. The C6‑chloro substituent in **32** was displaced via nucleophilic aromatic substitution (SN_Ar_) with either methanolic ammonia or 2‑chlorobenzylamine to yield **33a** and **33b**, respectively. Deprotection of **33a** and **33b** with aqueous trifluoroacetic acid (90% v/v) provided **CPD3e** and **CPD3f**. **CPD3g** was prepared from **CPD3e** using the same method with **CPD3a**.

# 3. Experiment section

## 3.1 General chemistry

All commercial solvents and reagents were used without further purification unless otherwise specified. All reactions were monitored by thin layer chromatography (TLC), using silica gel plates with fluorescence F254 and UV light visualization. Flash column chromatography was performed on silica gel (300–400 mesh). ^1^H NMR and ^13^C NMR spectra were recorded on a Bruker AV-400. In reported spectral data, the format (δ) chemical shift was used with the following abbreviations: s = singlet, d = doublet, t = triplet, q = quartet, m = multiplet. Low resolution mass spectra were recorded on an Agilent 1200 HPLC MSD mass spectrometer (Santa Clara, CA, USA) and high resolution mass spectra were recorded on an ABI/Sciex QStar mass spectrometer (AB SCIEX, Boston, USA).

**3.1.1 *(2R,3R,4S,5R)-2-(6-amino-9H-purin-9-yl)-5-(hydroxymethyl)-4-methoxy-tetrahydrofuran-3-ol* (CPD1a)**

Compound **CPD1a** was prepared according to the reported method ^1^. ^1^H NMR (400 MHz, DMSO-*d*_6_) δ 8.36 (s, 1H), 8.15 (s, 1H), 7.37 (s, 2H), 5.88 (d, *J* = 6.3 Hz, 1H), 5.55 – 5.45 (m, 2H), 4.82 – 4.73 (m, 1H), 4.12 – 4.04 (m, 1H), 3.87 (dd, *J* = 5.0, 3.0 Hz, 1H), 3.74 – 3.64 (m, 1H), 3.62 – 3.51 (m, 1H), 3.42 (s, 3H). ^13^C NMR (101 MHz, DMSO-*d*_6_) δ 156.2, 152.5, 149.1, 139.9, 119.4, 88.1, 83.4, 80.0, 72.9, 61.7, 57.7. HR-ESI-MS: [M+H]^+^ calcd for C_11_H_16_N_5_O_4_, 282.1202, found 282.1197.

**3.1.2 *(2R,3R,4R,5R)-2-(6-amino-9H-purin-9-yl)-5-(hydroxymethyl)tetrahydrofuran-3,4-diol* (CPD1b)**

Compound **CPD1b** was prepared according to the reported method ^2^. ^1^H NMR (400 MHz, DMSO-*d*_6_) δ 8.27 (s, 1H), 8.15 (s, 1H), 7.33 (s, 2H), 5.93 – 5.81 (m, 3H), 4.77 (t, *J* = 5.7 Hz, 1H), 4.36 – 4.28 (m, 1H), 4.18 – 4.12 (m, 1H), 4.06 –4.02 (m, 1H), 3.80 – 3.70 (m, 1H), 3.69 – 3.62 (m, 1H). ^13^C NMR (101 MHz, DMSO-*d*_6_) δ 156.1, 152.4, 148.7, 139.7, 118.8, 89.5, 83.5, 80.9, 75.2, 59.5. HR-ESI-MS: [M+H]+ calcd for C10H14N5O4, 268.1046, found 268.1040.

**3.1.3 *(2R,3S,4R,5R)-2-(6-amino-9H-purin-9-yl)-4-fluoro-5-(hydroxymethyl)tetra-hydrofuran-3-ol* (CPD1c)**

Compound **CPD1c** was prepared according to the reported method ^3^. ^1^H NMR (400 MHz, DMSO-*d*_6_) δ 8.17 (s, 1H), 8.10 (s, 1H), 7.33 (s, 2H), 6.30 (d, *J* = 4.5 Hz, 1H), 5.94 (d, *J* = 2.7 Hz, 1H), 5.22 – 4.99 (m, 2H), 4.81 – 4.71 (m, 1H), 4.39 – 4.24 (m, 1H), δ 3.82 – 3.68 (m, 1H). ^13^C NMR (101 MHz, DMSO-*d*_6_) δ 156.1, 152.8, 149.2, 138.4 (d, *J* = 6.1 Hz), 118.8, 95.6 (d, *J* = 183.5 Hz), 88.7 (d, *J* = 2.2 Hz), 81.9 (d, *J* = 19.5 Hz), 77.5 (d, *J* = 26.5 Hz), 58.2 (d, *J* = 10.2 Hz). HR-ESI-MS: [M+H]^+^ calcd for C_10_H_13_FN_5_O_3_, 270.1002, found 270.1040.

**3.1.4 *(2R,3S,4R,5R)-5-(6-amino-9H-purin-9-yl)-2-(hydroxymethyl)-3-methyl-tetrahydrofuran-3,4-diol* (CPD1d)**

Compound **CPD1d** was prepared according to the reported method ^4^. ^1^H NMR (400 MHz, DMSO-*d*_6_) δ 8.34 (s, 1H), 8.12 (s, 1H), 7.39 (s, 2H), 5.91 (dd, *J* = 8.3, 3.4 Hz, 1H), 5.84 (d, *J* = 7.9 Hz, 1H), 5.41 (d, *J* = 6.7 Hz, 1H), 4.83 (s, 1H), 4.45 (t, *J* = 7.4 Hz, 1H), 3.89 (s, 1H), 3.70 – 3.62 (m, 1H), 3.60 – 3.50 (m, 1H), 1.31 (s, 3H). ^13^C NMR (101 MHz, DMSO-*d*_6_) δ 156.3, 152.1, 149.0, 140.6, 119.6, 88.4, 87.4, 76.8, 76.3, 61.4, 19.9. HR-ESI-MS: [M+H]^+^ calcd for C_11_H_16_N_5_O_4_, 282.1202, found 282.1200.

**3.1.5 *(2R,3S,4R,5R)-5-(6-amino-9H-purin-9-yl)-3-ethynyl-2-(hydroxymethyl)-tetrahydrofuran-3,4-diol* (CPD1e)**

Compound **CPD1e** was prepared according to the reported method ^5^. ^1^H NMR (400 MHz, DMSO-*d*_6_) δ 8.35 (s, 1H), 8.13 (s, 1H), 7.38 (s, 2H), 6.01 (s, 1H), 5.87 (dd, *J* = 14.1, 7.4 Hz, 2H), 5.63 (dd, *J* = 7.4, 4.1 Hz, 1H), 4.81 (t, *J* = 7.4 Hz, 1H), 4.00 (t, *J* = 3.2 Hz, 1H), 3.82 – 3.66 (m, 2H), 3.56 (s, 1H). ^13^C NMR (101 MHz, DMSO-*d*_6_) δ 156.2, 152.4, 149.1, 140.3, 119.4, 87.8, 86.7, 82.8 77.5, 77.0, 72.8, 61.9. HR-ESI-MS: [M+H]^+^ calcd for C_12_H_14_N_5_O_4_, 292.1046, found 292.1040.

**3.1.6 *(2R,3R,5S)-2-(6-amino-9H-purin-9-yl)-5-(((tert-butyldimethylsilyl)oxy)-methyl)tetrahydrofuran-3-ol* (1)**

To a mixture of cordycepin (2.0 g, 8.00 mmol, 1eq) and imidazole (1.08 g, 15.88 mmol, 2 eq) was added TBSCl (1.3 g, 8.67 mmol, 1.1 eq) at 0℃. The reaction was stirred at room temperature for 2 h. After the reaction finished, the mixture was poured into water, extracted with ethyl acetate, washed with water and brine, dried over anhydrous Na_2_SO_4_, concentrated to dryness, and purified by flash column chromatography to yield **1** (2.9 g, 99.3%) as white foam. ^1^H NMR (400 MHz, DMSO-*d*_6_) δ 8.28 (s, 1H), 8.14 (s, 1H), 7.26 (s, 2H), 5.90 (d, *J* = 1.8 Hz, 1H), 5.70 (d, *J* = 4.1 Hz, 1H), 4.57 – 4.52 (m, 1H), 4.44 – 4.34 (m, 1H), 3.91 (dd, *J* = 11.5, 3.2 Hz, 1H), 3.73 (dd, *J* = 11.5, 3.9 Hz, 1H), 2.31 – 2.23 (m, 1H), 1.94 – 1.86(m, 1H), 0.87 (s, 9H), 0.04 (s, 3H), 0.03 (s, 3H). LRMS (ESI^-^) m/z: calcd for C_16_H_28_N_5_O_3_Si [M+H]^+^, 366.5; found, 366.9

**3.1.7 *Tert-butyl (tert-butoxycarbonyl)(9-((2R,3R,5S)-3-((tert-butoxycarbonyl)-oxy)-5-(((tert-butyldimethylsilyl)oxy)methyl)tetrahydrofuran-2-yl)-9H-purin-6-yl)carbamate* (2)：**

To a solution of **1** (2.9 g, 7.93 mmol, 1 eq), triethylamine (4.85 g, 48.0 mmol, 6 eq) and 4-dimethylaminopyridine (195 mg，1.6 mmol，0.2 eq) in dichloromethane (20 mL) at 0℃ was added di-tert-butyl decarbonate (7.85 g, 36 mmol, 4.5 eq). The reaction was stirred at room temperature for 6 h, then concentrated to dryness. The residue was diluted with water and extracted with ethyl acetate. The combined organic extracts were washed sequentially with 1 N aqueous HCl and saturated aqueous NaCl, dried over anhydrous Na₂SO₄, and filtered. The filtrate was concentrated, and the residue was purified by flash column chromatography to give **2** (4.0 g, 75.7%) as white foam. ^1^H NMR (400 MHz, DMSO-*d*_6_) δ 8.86 (s, 1H), 8.78 (s, 1H), 6.27 (d, *J* = 1.8 Hz, 1H), 5.66 – 5.58 (m, 1H), 4.46 – 4.36 (m, 1H), 3.89 (dd, *J* = 11.6, 3.0 Hz, 1H), 3.73 (dd, *J* = 11.6, 4.2 Hz, 1H), 2.66 – 2.60 (m, 1H), 2.24 – 2.18 (m, 1H), 1.38 (s, 9H), 1.37 (s, 18H), 0.81 (s, 9H), -0.03 (s, 3H), -0.06 (s, 3H). LRMS (ESI^-^) m/z: calcd for C_31_H_51_N_5_O_9_Si [M+H]^+^, 666.9; found, 670.0

**3.1.8 *Tert-butyl (tert-butoxycarbonyl)(9-((2R,3R,5S)-3-((tert-butoxycarbonyl)-oxy)-5-(hydroxymethyl)tetrahydrofuran-2-yl)-9H-purin-6-yl)carbamate* (3)**

Compound **2** (4.0 g, 6.02 mmol, 1 eq) was dissolved in THF (40 mL) under nitrogen atmosphere at room temperature. TEA·3HF (968 mg, 6 mmol, 1 eq) was added into the solution. The reaction was stirred at 40℃ for 8 h, then concentrated under reduced pressure. The residue was diluted with water and extracted with ethyl acetate. The collected organic phase was washed sequentially with saturated NaHCO_3_ aqueous solution and saturated aqueous NaCl, dried over anhydrous Na₂SO₄, and filtered. The filtrate was concentrated, and the residue was purified by flash column chromatography to give **3** (2.9 g, 87.7%) as white foam. ^1^H NMR (400 MHz, DMSO-*d*_6_) δ 8.87 (s, 1H), 8.86 (s, 1H), 6.26 (d, *J* = 2.3 Hz, 1H), 5.62 – 5.56 (m, 1H), 5.07 (t, *J* = 5.5 Hz, 1H), 4.44 – 4.32 (m, 1H), 3.72 – 3.64 (m, 1H), 3.56 – 3.50 (m, 1H), 2.61 – 2.53 (m, 1H), 2.25 – 2.19 (m, 1H), 1.39 (s, 18H), 1.37 (s, 9H). LRMS (ESI^-^) m/z: calcd for C_25_H_38_N_5_O_9_ [M+H]^+^, 552.6; found, 552.4

**3.1.9 *Tert-butyl (tert-butoxycarbonyl)(9-((2R,3R)-3-((tert-butoxycarbonyl)oxy)-5,5-bis(hydroxymethyl)tetrahydrofuran-2-yl)-9H-purin-6-yl)carbamate* (4)**

Compound **3** (8.0 g, 14.52 mmol, 1.0 eq), EDCI (8.4 g, 43.75 mmol, 3.0 eq), and pyridinium trifluoroacetate (1.4 g, 7.25 mmol, 0.5 eq) were successively added to DMSO. The resulting mixture was stirred at room temperature for 8 h. Upon completion, the reaction mixture was poured into water and extracted with ethyl acetate. The combined organic layers were washed with saturated aqueous NaCl, dried over anhydrous sodium sulfate, filtered, and concentrated under reduced pressure to afford a yellow oil, which was used directly in the subsequent step without further purification;

The crude product was dissolved in a mixture of 1,4-dioxane and water (*v: v*=10:1, 80 mL: 8 mL). To this solution, aqueous formaldehyde solution (10.8 mL, 10.0 eq) and sodium hydroxide (870 mg, 21.75 mmol, 1.5 eq) were added sequentially. The reaction was stirred at room temperature for 4 h. After completion, the mixture was poured into water and extracted with ethyl acetate. The organic extracts were combined, washed with saturated aqueous sodium chloride, dried over anhydrous sodium sulfate, filtered, and concentrated to give a yellow oil, which was used immediately in the next step;

Under an ice-water bath, the crude product was dissolved in methanol (100 mL). Sodium borohydride (1.1 g, 29.0 mmol, 2.0 eq) was added portion-wise, and the mixture was stirred for 2 h. The reaction was quenched by careful addition into saturated aqueous ammonium chloride. The resulting mixture was extracted with ethyl acetate. The combined organic layers were washed with saturated aqueous NaCl, dried over anhydrous Na₂SO₄, filtered, and concentrated. The crude residue was purified by silica gel column chromatography to afford **4** (2.4 g, 23.7% yield over the above three steps) as a white solid. ^1^H NMR (500 MHz, DMSO-*d*_6_) δ 8.89 (s, 1H), 8.85 (s, 1H), 6.28 (d, *J* = 5.3 Hz, 1H), 5.66 – 5.59 (m, 1H), 5.19 (t, *J* = 5.6 Hz, 1H), 5.07 (t, *J* = 5.8 Hz, 1H), 3.55 (dd, *J* = 11.5, 5.2 Hz, 1H), 3.52 – 3.41 (m, 3H), 2.60 (dd, *J* = 13.3, 8.3 Hz, 1H), 2.28 (dd, *J* = 13.2, 7.2 Hz, 1H), 1.36 (s, 18H), 1.25 (s, 9H). LRMS (ESI^-^) m/z: calcd for C_26_H_40_N_5_O_10_ [M+H]^+^, 582.6; found, 582.3;

**3.1.10 *Tert-butyl (tert-butoxycarbonyl)(9-((2R,3R,5S)-3-((tert-butoxycarbonyl)-oxy)-5-(((tert-butyldiphenylsilyl)oxy)methyl)-5-(hydroxymethyl)tetrahydrofuran -2-yl)-9H-purin-6-yl)carbamate* (5)**

To a mixture of **4** (5.3 g, 6.47 mmol, 1 eq) and imidazole (1.1 g, 16.18 mmol, 2.5 eq) in N, N-dimethylformamide (50 mL) at 0℃ was dropwise added tert-butylchloro-diphenylsilane (2.1 g, 7.64 mmol, 1.2 eq). The reaction was stirred for 4 h at room temperature. Then the residue was diluted with water and extracted with ethyl acetate. The collected organic layers were washed sequentially with saturated NaHCO_3_ aqueous solution and saturated aqueous NaCl, dried over anhydrous Na₂SO₄, and filtered. The filtrate was concentrated, and the residue was purified by flash column chromatography to give **5** (2.7 g, 37.1%) as white solid. ^1^H NMR (400 MHz, DMSO-*d*_6_) δ 8.90 (s, 1H), 8.87 (s, 1H), 7.71 – 7.63 (m, 4H), 7.53 – 7.37 (m, 6H), 6.32 (d, *J* = 4.6 Hz, 1H), 5.68 (dd, *J* = 8.9, 4.4 Hz, 1H), 5.24 (t, *J* = 5.5 Hz, 1H), 3.73 (s, 2H), 3.62 (dd, *J* = 11.6, 5.5 Hz, 1H), 3.56 (dd, *J* = 11.5, 5.7 Hz, 1H), 2.73 (dd, *J* = 13.6, 8.1 Hz, 1H), 2.38 (dd, *J* = 13.6, 6.0 Hz, 1H), 1.38 (s, 18H), 1.27 (s, 9H), 1.04 (s, 9H). LRMS (ESI^-^) m/z: calcd for C_42_H_58_N_5_O_10_Si [M+H]^+^, 821.0; found, 820.7;

**3.1.11 *Tert-butyl (9-((2R,3R,5S)-5-((bis(4-methoxyphenyl)(phenyl)methoxy)-methyl)-3-((tert-butoxycarbonyl)oxy)-5-(((tert-butyldiphenylsilyl)oxy)methyl)-tetrahydrofuran-2-yl)-9H-purin-6-yl)(tert-butoxycarbonyl)carbamate* (6)**

To a solution of **5** (2.7 g, 3.3 mmol, 1 eq) in dry dichloromethane (30 mL) was added triethylamine (480 mg, 4.8 mmol, 2 eq) and DMTrCl (1.4 g, 3.6 mmol, 1.5 eq) at 0℃ under nitrogen atmosphere. The mixture was stirred for 4 h at room temperature, then concentrated to dryness. The residue was diluted with water and extracted with ethyl acetate. The combined organic extracts were washed sequentially with 1 N aqueous HCl and saturated aqueous NaCl, dried over anhydrous Na₂SO₄, and filtered. The filtrate was concentrated, and the residue was purified by flash column chromatography to give **6** (2.9 g, 78.7%) as white foam. ^1^H NMR (400 MHz, DMSO-*d*_6_) δ 8.78 (s, 1H), 8.76 (s, 1H), 7.67 – 7.59 (m, 2H), 7.59 – 7.52 (m, 2H), 7.53 – 7.35 (m, 4H), 7.35 – 7.24 (m, 4H), 7.19 – 7.02 (m, 7H), 6.76 – 6.66 (m, 4H), 6.38 (d, *J* = 2.8 Hz, 1H), 5.81 – 5.77 (m, 1H), 3.86 (d, *J* = 9.7 Hz, 1H), 3.78 (d, *J* = 9.9 Hz, 1H), 3.69 (s, 3H), 3.68 (s, 3H), 3.40 (d, *J* = 9.7 Hz, 1H), 3.18 (d, *J* = 9.8 Hz, 1H), 2.89 (dd, *J* = 14.4, 8.0 Hz, 1H), 2.43 (dd, *J* = 14.4, 2.9 Hz, 1H), 1.36 (s, 18H), 1.34 (s, 9H), 0.87 (s, 9H).

**3.1.12 *Tert-butyl (9-((2R,3R,5R)-5-((bis(4-methoxyphenyl)(phenyl)methoxy)-methyl)-3-((tert-butoxycarbonyl)oxy)-5-(hydroxymethyl)tetrahydrofuran-2-yl)-9H-purin-6-yl)(tert-butoxycarbonyl)carbamate* (7)**

To a solution of **6** (2.9 g, 2.6 mmol, 1 eq) in THF (30 mL) was added a 1.0 M solution of TBAF in THF (2.6 mL, 2.6 mmol, 1 eq). The reaction mixture was stirred for 2 h at room temperature, then concentrated under reduced pressure. The residue was purified via flash column to give **7** (2.1 g, 91.4%). ^1^H NMR (400 MHz, DMSO-*d*_6_) δ 8.75 (s, 1H), 8.70 (s, 1H), 7.37 – 7.28 (m, 2H), 7.24 – 7.12 (m, 7H), 6.85 – 6.77 (m, 4H), 6.33 (d, *J* = 4.2 Hz, 1H), 5.87 – 5.77 (m, 1H), 5.10 (t, *J* = 5.5 Hz, 1H), 3.72 (s, 3H), 3.72 (s, 3H), 3.61 – 3.49 (m, 2H), 3.23 (d, *J* = 9.8 Hz, 1H), 3.13 (d, *J* = 9.8 Hz, 1H), 2.72 (dd, *J* = 13.9, 8.4 Hz, 1H), 2.30 (dd, *J* = 13.9, 5.1 Hz, 1H), 1.34 (s, 18H), 1.31 (s, 9H). LRMS (ESI^-^) m/z: calcd for C_47_H_58_N_5_O_12_ [M+H]^+^, 885.0; found, 885.7;

**3.1.13** ***Tert-butyl (9-((2R,3R,5S)-5-((bis(4-methoxyphenyl)(phenyl)methoxy)-methyl)-3-((tert-butoxycarbonyl)oxy)-5-(fluoromethyl)tetrahydrofuran-2-yl)-9H-purin-6-yl)(tert-butoxycarbonyl)carbamate* (8b)**

To solution of **7** (300 mg, 0.34 mmol, 1 eq) and pyridine (42 mg, 0.53 mmol, 1.5 eq) in dichloromethane (100 mL) was added trifluoromethanesulfonic anhydride (115 mg, 0.41 mmol, 1.2 eq) at -15℃. The solution was stirred for 2h, then poured into saturated NaHCO_3_ aqueous solution. The mixture was extracted, washed, dried and concentrated to dryness. The residue was dissolved in THF (3 mL), followed by the addition of 1M TBAF solution in THF (0.34 mL, 0.34 mmol, 1 eq). The mixture was stirred for 16 h at room temperature, then concentrated under reduced pressure. The residue was purified via flash column to afford **8b** (190 mg, 63.1% yield over two steps) as white solid. ^1^H NMR (400 MHz, DMSO-*d*_6_) δ 8.75 (s, 1H), 8.72 (s, 1H), 7.37 – 7.26 (m, 2H), 7.26 – 7.14 (m, 7H), 6.87 – 6.69 (m, 4H), 6.41 (d, *J* = 3.6 Hz, 1H), 5.92 – 5.80 (m, 1H), 4.66 (s, 1H), 4.54 (s, 1H), 3.72 (s, 6H), 3.28 (d, *J* = 10.0 Hz, 1H), 3.14 (d, *J* = 9.7 Hz, 1H), 2.96 – 2.84 (m, 1H), 2.26 (dd, *J* = 13.9, 4.2 Hz, 1H), 1.35 (s, 18H), 1.33 (s, 9H). LRMS (ESI^-^) m/z: calcd for C_47_H_57_FN_5_O_11_ [M+H]^+^, 887.0; found, 886.7;

**3.1.14 *Tert-butyl (9-((2R,3R,5R)-5-((bis(4-methoxyphenyl)(phenyl)methoxy)-methyl)-3-((tert-butoxycarbonyl)oxy)-5-cyanotetrahydrofuran-2-yl)-9H-purin-6-yl)(tert-butoxycarbonyl)carbamate*** **(8d)**

Compound **7** (500 mg, 0.56 mmol, 1 eq), 1-ethyl-3-(3-dimethyllaminopropyl)-carbodiimide hydrochloride (325 mg, 1,70 mg, 3 eq), pyridine trifluoroacetate (54 mg, 0.28 mmol, 0.5 eq) were added into dimethyl sulfoxide. The mixture was stirred for 3h at room temperature, then poured into water, extracted, washed, dried, and concentrated to dryness. The residue was dissolved in anhydrous pyridine (2 mL), followed by the addition of hydroxylamine hydrochloride (60 mg, 0.86 mmol, 1.5 eq). The mixture was stirred for 2 h at room temperature, then concentrated in vacuo. The residue was dissolved in anhydrous acetonitrile (2 mL), followed by the addition of 1,1'-carbonyldiimidazole (270 mg, 1.67 mmol, 3 eq). The solution was heated to 50 ℃ and stirred for 6 h. After completion, the mixture was poured into water, extracted with ethyl acetate, washed with water and brine, dried over anhydrous Na_2_SO_4_, concentrated to dryness, and purified by flash column chromatography to yield **8d** (303 mg, 61.6% yield over 3 steps). ^1^H NMR (400 MHz, DMSO-*d*_6_) δ 8.76 (s, 1H), 8.69 (s, 1H), 7.31 – 7.28 (m, 2H), 7.20 – 7.12 (m, 7H), 6.86 – 6.75 (m, 3H), 6.68 (d, *J* = 1.7 Hz, 1H), 5.78 – 5.71 (m, 1H), 3.72 (s, 3H), 3.71 (s, 3H), 3.50 (d, *J* = 9.9 Hz, 1H), 3.38 (d, *J* = 9.9 Hz, 1H), 3.23 (dd, *J* = 15.1, 6.9 Hz, 1H), 2.87 – 2.78 (m, 1H), 1.41 (s, 9H), 1.37 (s, 18H). LRMS (ESI^-^) m/z: calcd for C_47_H_55_N_6_O_11_ [M+H]^+^, 879.0; found, 879.3;

**3.1.15 General procedure A** for the synthesis of compounds **CPD1f~CPD1j**

Compounds **4**, **8a**~**8d** were added into a solution of trifluoroacetic acid and dichloromethane (*v: v*=1:4, 2 mL). The reaction was stirred for 5 h at room temperature, then concentrated to dryness. The residue was purified by flash column chromatography to yield the target compounds.

**3.1.16** ***((4R,5R)-5-(6-amino-9H-purin-9-yl)-4-hydroxytetrahydrofuran-2,2-diyl)-dimethanol*** **(CDP1f)**

Compound **CDP1f** (77 mg, 80.6% yeild) was obtained from intermediate **4** (200 mg, 0.34 mmol, 1 eq) using **General procedure A**. ^1^H NMR (400 MHz, DMSO-*d*_6_) δ 8.32 (s, 1H), 8.13 (s, 1H), 7.34 (s, 2H), 5.79 (d, *J* = 6.0 Hz, 1H), 5.58 (d, *J* = 5.5 Hz, 1H), 5.50 (dd, *J* = 8.0, 4.2 Hz, 1H), 5.02 (t, *J* = 5.7 Hz, 1H), 4.87 – 4.75 (m, 1H), 3.49 (dd, *J* = 11.7, 3.8 Hz, 1H), 3.45 – 3.34 (m, 3H), 2.33 (dd, *J* = 12.6, 7.7 Hz, 1H), 2.01 (dd, *J* = 12.6, 8.1 Hz, 1H). ^13^C NMR (101 MHz, DMSO-*d*_6_) δ 156.2, 152.3, 149.0, 140.0, 119.4, 90.3, 87.3, 73.5, 64.8, 64.5, 36.0. HR-ESI-MS: [M+H]^+^ calcd for C_11_H_16_N_5_O_4_, 282.1202, found 282.1210.

**3.1.17** ***(2R,3R,5S)-2-(6-amino-9H-purin-9-yl)-5-(hydroxymethyl)-5-methyltetra-hydrofuran-3-ol*** **(CPD1g):**

Compound **7** (300 mg, 0.34 mmol, 1 eq) and pyridine (50 mg, 0.51 mmol, 1.5 eq) was added in dichloromethane (5 mL), followed by slow addition of trifluoromethanesulfonic anhydride (115 mg, 0.41 mmol, 1.2 eq) at -20℃ under nitrogen atmosphere. The reaction mixture was stirred for 5 h, then poured into saturated NaHCO_3_ aqueous solution, extracted with dichloromethane, washed with water and brine, dried over anhydrous Na_2_SO_4_, concentrated to dryness. The residue was dissolved in toluene, followed by addition of tetrabutylammonium borohydride (355 mg, 1.38 mmol, 4 eq). The reaction was stirred at 85℃ for 8 h, then cooled to room temperature. The solution was poured into ice-water, extracted with ethyl acetate, washed with water and brine, dried over anhydrous Na_2_SO_4_, concentrated to dryness. The residue **8a** was directly used without purification. Compound **CPD1g** (64 mg, 71.0% yield) was obtained from intermediate **8a** (200 mg, 0.34 mmol, 1 eq) using **General procedure A**. ^1^H NMR (400 MHz, DMSO-*d*_6_) δ 8.33 (s, 1H), 8.13 (s, 1H), 7.33 (s, 2H), 5.83 (d, *J* = 5.5 Hz, 1H), 5.57 (dd, *J* = 11.1, 4.5 Hz, 2H), 4.87 – 4.77 (m, 1H), 3.45 (dd, *J* = 11.5, 4.3 Hz, 1H), 3.29 (dd, *J* = 11.6, 7.9 Hz, 1H), 2.54 - 2.52 (m, 1H), 1.79 (dd, *J* = 12.7, 7.3 Hz, 1H), 1.27 (s, 3H). ^13^C NMR (101 MHz, DMSO-*d*_6_) δ 156.2, 152.3, 148.9, 139.9, 119.3, 90.1, 84.8, 74.0, 68.2, 40.8, 24.8. HR-ESI-MS: [M+H]^+^ calcd for C_11_H_16_N_5_O_3_, 266.1253, found 266.1217.

**3.1.18** ***(2R,3R,5S)-2-(6-amino-9H-purin-9-yl)-5-(fluoromethyl)-5-(hydroxymethyl) tetrahydrofuran-3-ol* (CPD1h)**

Compound **CPD1h** (33 mg, 58.3% yeild) was obtained from intermediate **8b** (183 mg, 0.20 mmol, 1 eq) using **General procedure A**. ^1^H NMR (400 MHz, DMSO-*d*_6_) δ 8.34 (s, 1H), 8.14 (s, 1H), 7.35 (s, 2H), 7.11 (s, 1H), 5.84 (d, *J* = 5.6 Hz, 1H), 5.69 (d, *J* = 4.8 Hz, 1H), 5.50 (s, 1H), 4.93 – 4.84 (m, 1H), 4.58 – 4.46 (m, 1H), 4.46 – 4.34 (m, 1H), 3.57 (d, *J* = 11.4 Hz, 1H), 3.45 (d, *J* = 11.6 Hz, 1H), 2.48 – 2.42 (m, 1H), 1.89 (dd, *J* = 13.1, 7.5 Hz, 1H). ^13^C NMR (101 MHz, DMSO-*d*_6_) δ 156.2, 152.4, 149.1, 139.9, 119.3, 90.0, 85.2 (d, *J* = 17.3 Hz), 84.7 (d, *J* = 172.1 Hz), 72.8, 63.1 (d, *J* = 5.4 Hz), 35.2 (d, *J* = 4.0 Hz). HR-ESI-MS: [M+H]^+^ calcd for C_11_H_15_FN_5_O_3_, 284.1159, found 284.1158.

**3.1.19** ***(2R,3R,5S)-2-(6-amino-9H-purin-9-yl)-5-(chloromethyl)-5-(hydroxymethyl) tetrahydrofuran-3-ol* (CPD1i)**:

Compound **7** (304 mg, 0.34 mmol, 1 eq) was reacted with trifluoromethanesulfonic anhydride (120mg, 0.41 mmol, 1.2 eq) under the same condition with **8a**. The obtained triflate was dissolved in DMF (3 mL). Lithium chloride (71 mg, 1.69 mmol, 5 eq) was added into the solution, and stirred for 16 h at 40℃. Then the mixture was poured into water, extracted with ethyl acetate, washed with water and brine, dried over anhydrous Na_2_SO_4_, concentrated to dryness. The residue **8c** was directly used without purification. Compound **CPD1i** (37 mg, 61.8%) was obtained from intermediate **8c** (183 mg, 0.20 mmol, 1 eq) using **General procedure A**. ^1^H NMR (400 MHz, DMSO-*d*_6_) δ 8.34 (s, 1H), 8.15 (s, 1H), 7.36 (s, 2H), 5.89 (d, *J* = 5.6 Hz, 1H), 5.72 (d, *J* = 4.8 Hz, 1H), 5.62 (s, 1H), 4.94 – 4.84 (m, 1H), 3.87 – 3.74 (m, 2H), 3.64 – 3.56 (m, 1H), 3.53 – 3.45 (m, 1H), 2.49 – 2.45 (m, 1H), 2.06 (dd, *J* = 13.1, 7.5 Hz, 1H). ^13^C NMR (101 MHz, DMSO-*d*_6_) δ 156.2, 152.4, 149.0, 139.9, 119.3, 90.3, 85.7, 73.2, 64.5, 48.5, 37.2. HR-ESI-MS: [M+H]^+^ calcd for C_11_H_15_ClN_5_O_3_, 300.0863, found 300.1055.

**3.1.20 *(2R,4R,5R)-5-(6-amino-9H-purin-9-yl)-4-hydroxy-2-(hydroxymethyl)tetra-hydrofuran-2-carbonitrile* (CPD1j)**

Compound **CPD1i** (24 mg, 25.6% yeild) was obtained from intermediate **8d** (300 mg, 0.34 mmol, 1 eq) using **General procedure A**. ^1^H NMR (400 MHz, DMSO-*d*_6_) δ 8.3 (s, 1H), 8.2 (s, 1H), 7.4 (s, 2H), 6.1 (d, *J* = 2.7 Hz, 1H), 6.0 (d, *J* = 3.7 Hz, 1H), 5.9 (t, *J* = 6.2 Hz, 1H), 4.9 – 4.8 (m, 1H), 3.8 (dd, *J* = 11.9, 5.0 Hz, 1H), 3.7 (dd, *J* = 11.8, 5.4 Hz, 1H), 2.9 (dd, *J* = 13.9, 6.1 Hz, 1H), 2.4 (dd, *J* = 13.8, 2.9 Hz, 1H). ^13^C NMR (101 MHz, DMSO-*d*_6_) δ 156.1, 152.7, 149.0, 139.5, 120.1, 119.0, 91.6, 80.4, 73.4, 65.2, 40.2. HR-ESI-MS: [M+H]^+^ calcd for C11H13N6O3, 277.1049, found 277.1000.

**3.1.21** ***((2S,4R,5R)-4-acetoxy-5-(2,6-dichloro-9H-purin-9-yl)tetrahydrofuran-2-yl)methyl benzoate*** **(10a):**

To a solution of 2,6-dichloropurine (1.1 g, 5.9 mmol, 1 eq) in acetonitrile was added N, O-bis(trimethylsilyl)acetamide (2 eq) under nitrogen atmosphere. The mixture was stirred at 80℃ for 1 hour, then cooled to room temperature. Intermediate **9** (1.9 g, 5.9 mmol, 1 eq) and trimethylsilyl trifluoromethanesulfonate (2 eq) was added at room temperature and stirred at 80℃ for 2 hours. After the reaction was finished, the reaction mixture was poured into saturated NaHCO_3_ aqueous solution, extracted with ethyl acetate, washed with water and brine, dried over anhydrous Na_2_SO_4_, concentrated to dryness, and purified by flash column chromatography to yield **10a** (1.8 g, 67.6% yield, white foam) as white foam, respectively. ^1^H NMR (400 MHz, DMSO-*d*_6_) δ 8.85 (s, 1H), 7.87 – 7.80 (m, 2H), 7.69 – 7.60 (m, 1H), 7.54 – 7.43 (m, 2H), 6.27 (d, *J* = 1.4 Hz, 1H), 5.82– 5.74 (m, 1H), 4.73 - 4.67 (m, 1H), 4.61 (dd, *J* = 12.2, 2.9 Hz, 1H), 4.49 (dd, *J* = 12.2, 5.6 Hz, 1H), 2.76 – 2.68 (m, 1H), 2.38 – 2.30 (m, 1H), 2.12 (s, 3H). LRMS (ESI^-^) m/z: calcd for C_19_H_17_Cl_2_N_4_O_5_ [M+H]^+^, 452.3; found, 452.5;

**3.1.22 *(2R,3R,4R,5R)-2-(acetoxymethyl)-5-(2,6-dichloro-9H-purin-9-yl)tetra hydrofuran-3,4-diyl diacetate* (10b)**

Compound **10b** (2.5 g, 52.8% yield) was prepared from 2,6-dichloropurine (2.0 g, 10.6 mmol, 1 eq) and **9b** (3.4 g, 10.6 mmol, 1 eq) using the same method used for **10a**. ^1^H NMR (400 MHz, DMSO-*d*_6_) δ 8.9 (s, 1H), 6.3 (d, *J* = 4.9 Hz, 1H), 5.9 (t, *J* = 5.5 Hz, 1H), 5.6 (t, *J* = 5.6 Hz, 1H), 4.5 – 4.4 (m, 2H), 4.3 (dd, *J* = 12.1, 5.4 Hz, 1H), 2.1 (s, 3H), 2.1 (s, 3H), 2.0 (s, 3H). LRMS (ESI^-^) m/z: calcd for C_16_H_17_Cl_2_N_4_O_7_ [M+H]^+^, 448.2; found, 448.1;

**3.1.23 General procedure B** for the synthesis of compound **CPD2a~CPD2e:** To a solution of **10a** or **10b** (200 mg, 1 eq) and triethylamine (2 eq) in ethanol (2 mL) was added an appropriate amine (1.5 eq) under N_2_ at room temperature. The mixture was heated to 60 ℃ and stirred for 16 hours. After completion of the reaction, the mixture was concentrated under reduced pressure to obtain the crude product **11a~11e**, which was directly used in the next reaction. The crude products **11a~11e** and 7N methanolic ammonia (5 mL) were added into a 15 mL sealed tube, and stirred for 12 h at 50℃. After cooling to room temperature, the mixture was concentrated under reduced pressure. The residue was purified through flash column to obtain **CPD2a~CPD2e.**

**3.1.24 *(2R,3R,4S,5R)-2-(2-chloro-6-((2-chlorobenzyl)amino)-9H-purin-9-yl)-5-(hydroxymethyl)tetrahydrofuran-3,4-diol* (CPD2a)**

Compound **CPD2a** (37 mg, 39.5% yield over two-step) was prepared from **10b** (100 mg, 0.22 mmol, 1 eq) and 2-chlorobenzylamine (49 mg, 0.33 mmol, 1.5 eq) using the **General procedure B**. ^1^H NMR (400 MHz, DMSO-*d*_6_) δ 8.92 (t, *J* = 6.3 Hz, 1H), 8.47 (s, 1H), 7.49 – 7.42 (m, 1H), 7.32 – 7.25 (m, 3H), 5.85 (d, *J* = 5.9 Hz, 1H), 5.50 (d, *J* = 5.9 Hz, 1H), 5.22 (d, *J* = 4.9 Hz, 1H), 5.06 (t, *J* = 6.0 Hz, 1H), 4.79 – 4.64 (m, 2H), 4.59 – 4.48 (m, 1H), 4.17 – 4.12 (m, 1H), 3.99 – 3.93 (m, 1H), 3.73 – 3.61 (m, 1H), 3.61 – 3.51 (m, 1H). ^13^C NMR (101 MHz, DMSO-*d*_6_) δ 155.1, 153.1, 149.8, 140.3, 136.0, 131.9, 129.2, 128.6, 128.3, 127.2, 118.7, 87.5, 85.8, 73.7, 70.4, 61.4, 41.2. HR-ESI-MS: [M+H]^+^ calcd for C_17_H_18_Cl_2_N_5_O_4_, 425.0658, found 425.0666.

**3.1.25 *(2R,3R,5S)-2-(6-(benzylamino)-2-chloro-9H-purin-9-yl)-5-(hydroxymethyl) tetrahydrofuran-3-ol* (CPD2b)**

Compound **CPD2b** (41 mg, 33.3% yield over two-step) was prepared from **10a** (150 mg, 0.33 mmol, 1 eq) and benzylamine (55 mg, 0.50 mmol, 1.5 eq) using the **General procedure B**. ^1^H NMR (400 MHz, DMSO-*d*_6_) δ 8.87 (t, *J* = 6.7 Hz, 1H), 8.42 (s, 1H), 7.37 – 7.27 (m, 4H), 7.27 – 7.19 (m, 1H), 5.82 (s, 1H), 5.68 (d, *J* = 4.0 Hz, 1H), 5.01 (t, *J* = 5.5 Hz, 1H), 4.71 – 4.57 (m, 1H), 4.55 – 4.51 (m, 1H), 4.40 – 4.32 (m, 1H), 3.75 – 3.65 (m, 1H), 3.54 (dd, *J* = 12.0, 6.3 Hz, 1H), 2.28 – 2.14 (m, 1H), 1.95 – 1.85 (m, 1H). ^13^C NMR (101 MHz, DMSO-*d*_6_) δ 154.9, 153.0, 149.2, 139.4, 139.2, 128.3, 127.3, 126.8, 118.5, 90.7, 81.0, 74.8, 62.3, 43.1, 33.8. HR-ESI-MS: [M + H]^+^ calcd for C_17_H_19_ClN_5_O_3_, 376.1176, found 376.1172.

**3.1.26 *(2R,3R,5S)-2-(2-chloro-6-((2-chlorobenzyl)amino)-9H-purin-9-yl)-5-(hydroxymethyl)tetrahydrofuran-3-ol* (CPD2c)**

Compound **CPD2c** (41 mg, 33.3% yield over two-step) was prepared from **10a** (150 mg, 0.33 mmol, 1 eq) and benzylamine (55 mg, 0.50 mmol, 1.5 eq) using the **General procedure B**. ^1^H NMR (400 MHz, DMSO-*d*_6_) δ 8.87 (t, *J* = 6.3 Hz, 1H), 8.46 (s, 1H), 7.49 – 7.42 (m, 1H), 7.35 – 7.25 (m, 3H), 5.84 (s, 1H), 5.69 (d, *J* = 4.2 Hz, 1H), 5.03 (t, *J* = 5.6 Hz, 1H), 4.75 – 4.68 (m, 1H), 4.54 (s, 1H), 4.37 (dd, *J* = 9.7, 5.5 Hz, 1H), 3.76 – 3.66 (m, 1H), 3.59 – 3.49 (m, 1H), 2.29 – 2.17 (m, 1H), 1.95 –1.85 (m, 1H). ^13^C NMR (101 MHz, DMSO-*d*_6_) δ 155.0, 153.0, 149.4, 139.6, 136.0, 131.9, 129.2, 128.6, 128.3, 127.2, 118.6, 90.7, 81.1, 74.9, 62.3, 33.8. HR-ESI-MS: [M+H]^+^ calcd for C_17_H_18_Cl_2_N_5_O_3_, 410.0787, found 410.0806.

**3.1.27** ***(2R,3R,5S)-2-(2-chloro-6-((2,4-dichlorobenzyl)amino)-9H-purin-9-yl)-5-(hydroxymethyl)tetrahydrofuran-3-ol* (CPD2d)**

Compound **CPD2d** (53 mg, 36.3% yield over two-step) was prepared from **10a** (150 mg, 0.33 mmol, 1 eq) and 2,4-dichlorobenzylamine (88 mg, 0.50 mmol, 1.5 eq) using the **General procedure B**. ^1^H NMR (400 MHz, DMSO-*d*_6_) δ 8.90 (t, *J* = 6.0 Hz, 1H), 8.47 (s, 1H), 7.61 (d, *J* = 2.0 Hz, 1H), 7.54 – 7.07 (m, 2H), 5.84 (s, 1H), 5.70 (d, *J* = 4.1 Hz, 1H), 5.03 (t, *J* = 5.5 Hz, 1H), 4.68 (dd, *J* = 6.1, 3.1 Hz, 1H), 4.54 (s, 1H), 4.42 – 4.33 (m, 1H), 3.75 – 3.65 (m, 1H), 3.59 – 3.49 (m, 1H), 2.29 – 2.17 (m, 1H), 1.96 – 1.86 (m, 1H). ^13^C NMR (101 MHz, DMSO-*d*_6_) δ 171.5, 154.9, 153.0, 149.4, 139.7, 135.3, 132.9, 132.2, 129.8, 128.6, 127.3, 118.6, 90.7, 81.1, 74.9, 62.3, 33.8. HR-ESI-MS: [M+H]^+^ calcd for C_17_H_17_Cl_3_N_5_O_3_, 444.0397, found 444.0521.

**3.1.28** ***(2R,3R,5S)-2-(2-chloro-6-((2-chloro-4-fluorobenzyl)amino)-9H-purin-9-yl)-5-(hydroxymethyl)tetrahydrofuran-3-ol* (CPD2e)**

Compound **CPD2e** (45 mg, 31.9% yield over two-step) was prepared from **10a** (150 mg, 0.33 mmol, 1 eq) and 2,4-dichlorobenzylamine (80 mg, 0.50 mmol, 1.5 eq) using the **General procedure B**. ^1^H NMR (400 MHz, DMSO-*d*_6_) δ 8.88 (t, *J* = 6.0 Hz, 1H), 8.46 (s, 1H), 7.45 (dd, *J* = 8.8, 2.6 Hz, 1H), 7.38 – 7.32 (m, 1H), 7.21 – 7.13 (m, 1H), 5.84 (d, *J* = 2.1 Hz, 1H), 5.69 (d, *J* = 4.1 Hz, 1H), 5.03 (t, *J* = 5.6 Hz, 1H), 4.67 (t, *J* = 4.5 Hz, 1H), 4.54 (s, 1H), 4.42 – 4.32 (m, 1H), 3.75 – 3.65 (m, 1H), 3.59 – 3.49 (m, 1H), 2.26 – 2.20 (m, 1H), 1.94 – 1.86 (m, 1H). ^13^C NMR (101 MHz, DMSO-*d*_6_) δ 162.17, 159.73, 154.88, 152.98, 149.38, 139.66, 132.62 (d, *J* = 10.4 Hz), 132.38, 130.01 (d, *J* = 9.0 Hz), 118.59, 116.46 (d, *J* = 25.0 Hz), 114.26 (d, *J* = 21.1 Hz), 90.70, 81.07, 74.86, 62.26, 33.78. HR-ESI-MS: [M+H]^+^ calcd for C_17_H_17_Cl_2_FN_5_O_3_, 428.0692, found 428.0690.

**3.1.29 General procedure C** for the synthesis of **CPD3a** and **CPD3b**

Nucleoside **12a** or **12b** (1 eq) was suspended in a mixed solution of acetonitrile and water (*v: v*=500:1, 20V). 2-Acetoxyisobutyryl bromide (4eq) was added into the mixture at 0 ℃ and stirred at room temperature for 8 h. After completion, saturated NaHCO_3_ aqueous solution was slowly added into the suspension. The reaction mixture was extracted with ethyl acetate, washed with water and brine, dried over anhydrous Na_2_SO_4_, concentrated to dryness. The residue was dissolved in methanol, then NaOMe (4eq) was added into the solution. The mixture was stirred for 8 h at room temperature, then filtered to remove insoluble solid. The filtrate was concentrated under reduced pressure and purified by flash column chromatography to yield **13a** and **13b**, respectively.

Compound **13a** and **13b** (1 eq) was suspended in anhydrous THF under nitrogen atmosphere. 1.0 M lithium triethylborohydride in THF (10 eq) was added into the mixture at 0 ℃ and stirred for 16 h at room temperature. After completion, the reaction was quenched by the careful dropwise addition of 30% hydrogen peroxide in H_2_O. The mixture was concentrated in vacuo. The residue was purified by flash column chromatography to give **CPD3a** and **CPD3b**.

**3.1.30** ***(2S,3R,5S)-2-(4-aminoimidazo[2,1-f][1,2,4]triazin-7-yl)-5-(hydroxymethyl) tetrahydrofuran-3-ol* (CPD3a)**

Compound **CDP3a** (white foam, 1.7 g, 45.3%) was obtained from intermediate **12a** (4 g, 15.0 mmol, 1 eq) using **General procedure C**. ^1^H NMR (400 MHz, DMSO-*d*_6_) δ 7.83 (s, 1H), 6.82 (d, *J* = 4.4 Hz, 1H), 6.63 (d, *J* = 4.4 Hz, 1H), 5.21 (d, *J* = 4.5 Hz, 1H), 5.11 (d, *J* = 3.3 Hz, 1H), 4.72 (t, *J* = 5.7 Hz, 1H), 4.41 – 4.33 (m, 1H), 4.23 – 4.15 (m, 1H), 3.56 – 3.40 (m, 2H), 1.96 – 1.90 (m, 1H), 1.86 – 1.80 (m, 1H). ^13^C NMR (101 MHz, DMSO-*d*_6_) δ 156.05, 148.14, 130.28, 115.04, 109.56, 101.18, 80.19, 79.60, 64.27, 37.26. HR-ESI-MS: [M+H]^+^ calcd for C_11_H_15_N_4_O_3_, 251.1144, found 251.1140.

**3.1.31** ***(2S,3R,5S)-2-(4-aminopyrrolo[2,1-f][1,2,4]triazin-7-yl)-5-(hydroxymethyl) tetrahydrofuran-3-ol* (CPD3b)**

Compound **CDP3b** (white solid, 313 mg, 41.6% yeild) was obtained from intermediate **12b** (800 g, 3.0 mmol, 1 eq) using **General procedure C**. ^1^H NMR (400 MHz, DMSO-*d*_6_) δ 8.22 – 8.08 (m, 2H), 8.07 (s, 1H), 7.59 (s, 1H), 5.32 (s, 1H), 5.03 (d, *J* = 3.1 Hz, 1H), 4.74 (s, 1H), 4.48 – 4.42 (m, 1H), 4.26 – 4.16 (m, 1H), 3.54 (dd, *J* = 11.7, 3.9 Hz, 1H), 3.47 (dd, *J* = 11.7, 4.7 Hz, 1H), 2.01 – 1.95 (m, 1H), 1.88 – 1.82 (m, 1H). ^13^C NMR (101 MHz, DMSO-*d*_6_) δ 154.0, 149.0, 129.6, 129.2, 128.7, 79.4, 78.7, 74.0, 63.5, 36.4. HR-ESI-MS: [M+H]^+^ calcd for C_10_H_14_N_5_O_3_, 252.1097, found 252.1107.

**3.1.32** ***Tert-butyldiphenyl(((3aR,5S,6aR)-2,2,6a-trimethyltetrahydrofuro[2,3-d] [1,3]dioxol-5-yl)methoxy)silane* (16)**

To solution of **15** (9.4 g, 21.27 mmol, 1 eq) and pyridine (3.1 g, 31.63 mmol, 1.5 eq) in dichloromethane (100 mL) was added trifluoromethanesulfonic anhydride (6.7 g, 25.57 mmol, 1.2 eq) at -15℃. The solution was stirred for 2h, then poured into saturated NaHCO_3_ aqueous solution. The mixture was extracted, washed, dried and concentrated to dryness. The residue was dissolved in toluene (200 mL), then tetrabutylammonium borohydride (16.4 g, 63.81 mmol, 3 eq) was added to reaction. The reaction was stirred at 85℃ for 16 h, then cooled to room temperature. The mixture was poured into ice-water, extracted with ethyl acetate, washed with water and brine, dried over anhydrous Na_2_SO_4_, concentrated to dryness, and purified by flash column chromatography to yield **16** (7.2 g, 79.5% yield over two steps) as colorless oil. ^1^H NMR (400 MHz, DMSO-*d*_6_) δ 7.66 – 7.60 (m, 4H), 7.49 – 7.40 (m, 6H), 5.43 (s, 1H), 4.32 – 4.22 (m, 1H), 3.74 (dd, *J* = 11.1, 3.7 Hz, 1H), 3.67 (dd, *J* = 11.1, 4.6 Hz, 1H), 2.04 (dd, *J* = 13.1, 5.0 Hz, 1H), 1.72 (dd, *J* = 13.0, 10.4 Hz, 1H), 1.41 (s, 3H), 1.37 (s, 3H), 1.33 (s, 3H), 1.00 (s, 9H). LRMS (ESI^-^) m/z: calcd for C_25_H_35_O_4_Si [M+H]^+^, 427.6; found, 427.5;

**3.1.33** ***(2S,3R,5S)-3-(benzyloxy)-5-((benzyloxy)methyl)-3-methyltetrahydrofuran-2-ol* (19)**

To a solution of **16** (6.6 g, 15.49 mmol, 1 eq) in methanol (50 mL) was dropwise added conc. H_2_SO_4_ (0.5 mL) at 0℃. The solution was stirred at room temperature for 4 h, then concentrated in vacuo. The residue was partitioned between H_2_O and EtOAc. The collected organic layers were washed with saturated NaHCO_3_ aqueous solution and brine, dried over Na_2_SO_4_, and concentrated to give the crude product **17**. Crude **17** was dissolved in anhydrous THF, followed by the addition of 60% NaH (1.18 g, 29.5 mmol, 2 eq) at 0℃. The solution was stirred for 20 minutes, then tetrabutylammonium iodide (540 mg, 1.4 mmol, 0.1 eq) and benzyl bromide (5.04 g, 29.5 mmol, 2 eq) was added to the reaction. After stirring at 70 °C for 16 hours, the reaction mixture was cooled to room temperature and slowly poured into saturated aqueous NH₄Cl solution. The mixture was extracted with ethyl acetate, washed with water and brine, dried over anhydrous Na_2_SO_4_, concentrated to obtained the crude product **18**. Crude **18** was dissolved in 80% AcOH in water (30 mL) and stirred at 80℃ for 6 h. After cooling to room temperature, the mixture was concentrated in vacuo. The residue was purified via flash column to yield **19** (2.3 g, 45.3% yield over three steps) as colorless oil. ^1^H NMR (400 MHz, DMSO-*d*_6_) δ 7.38 – 7.29 (m, 10H), 6.25 (d, *J* = 5.0 Hz, 1H), 4.97 (d, *J* = 5.1 Hz, 1H), 4.51 (s, 2H), 4.45 (d, *J* = 4.0 Hz, 2H), 4.26 – 4.15 (m, 1H), 3.55 – 3.41 (m, 2H), 2.21 (dd, *J* = 13.1, 6.0 Hz, 1H), 1.56 (dd, *J* = 13.2, 9.8 Hz, 1H). LRMS (ESI^-^) m/z: calcd for C_20_H_25_O_4_ [M+H]^+^, 329.4; found, 329.3;

**3.1.34** ***(3R,5S)-3-(benzyloxy)-5-((benzyloxy)methyl)-3-methyldihydrofuran-2(3H)-one* (20)**

To a solution of **19** (2.2 g, 6.71 mmol, 1 eq) in DMSO (6 mL) was added acetic anhydride (4 mL) at room temperature. After stirring for 48 h, the mixture was poured into water, extracted with ethyl acetate, washed with water and brine, dried over anhydrous Na_2_SO_4_, concentrated to dryness, and purified by flash column chromatography to yield **20** (2.0 g, 91.4%) as white solid. ^1^H NMR (400 MHz, DMSO-*d*_6_) δ 7.38 – 7.24 (m, 10H), 4.83 – 4.74 (m, 1H), 4.55 (d, *J* = 1.5 Hz, 2H), 4.52 – 4.42 (m, 2H), 3.68 (dd, *J* = 11.3, 2.8 Hz, 1H), 3.56 (dd, *J* = 11.3, 5.8 Hz, 1H), 2.49 – 2.45 (m, 1H), 2.12 – 2.02 (m, 1H), 1.46 (s, 3H). LRMS (ESI^-^) m/z: calcd for C_20_H_23_O_4_ [M+H]^+^, 327.4; found, 327.5;

**3.1.35** ***7-((2S,3R,5S)-3-(benzyloxy)-5-((benzyloxy)methyl)-3-methyltetrahydrofuran-2-yl)pyrrolo[2,1-f][1,2,4]triazin-4-amine* (22)**

7-Iodopyrrolo[2,1-f][1,2,4]triazin-4-amine (1.57 g, 6.13 mmol, 1 eq) was suspended in THF under atmosphere. Chlorotrimethylsilane (1.34 g, 12.26 mmol, 2 eq) were added, and the mixture was stirred for 20 min at room temperature. The reaction was cooled to 0°C, 2.0 M PhMgCl in THF (6.1 mL, 12.2 mmol, 2 eq) was added dropwise. The mixture was stirred for another 20 min after which 2.0 M iPrMgCl in THF (3.7 mL, 6.4 mmol, 1.2 eq) was added slowly. The mixture was stirred at room temperature for 1 h, then cooled to -15°C. A solution of **20** (2.0 g, 6.13 mmol, 1 eq) was added dropwise to the reaction. After stirring for 4 h, the reaction solution was poured into saturated NH_4_Cl aqueous solution, extracted with ethyl acetate, washed with water and brine, dried over anhydrous Na_2_SO_4_, and concentrated under reduced pressure. The residue was purified by flash column chromatography to yield **21** (1.84 g, 65.3%) as yellow oil. LRMS (ESI^-^) m/z: calcd for C_26_H_28_N_4_O_4_ [M+H]^+^, 460.5; found, 460.4;

To a solution of **21** (500 mg, 1.09 mmol, 1 eq) in anhydrous dichloromethane (5 mL) was added triethylsilane (630 mg, 5.50 mmol, 5 eq) and 46.5% boron trifluoride ether solution (0.86 mL, 3 eq) at 0°C. After stirring at 0°C for 4 h, the reaction mixture was poured into saturated aqueous NaHCO_3_ solution. The mixture was extracted with dichloromethane, washed with water and brine, dried over anhydrous Na_2_SO_4_, and concentrated to dryness. The residue was purified by flash column chromatography to yield **22** (295 mg, 69.9%) as white foam. LRMS (ESI^+^) m/z: calcd for C_25_H_35_N_5_O_10_[M+23]^+^, 445.3; found, 467.3；^1^H NMR (400 MHz, DMSO-*d*_6_) δ 7.84 (s, 1H), 7.67 (s, 2H), 7.41 – 7.25 (m, 10H), 6.88 – 6.76 (m, 1H), 6.68 (d, *J* = 4.3 Hz, 1H), 5.62 (s, 1H), 4.69 – 4.54 (m, 5H), 4.41 – 4.33 (m, 1H), 3.76 (dd, *J* = 10.6, 3.4 Hz, 1H), 3.71 – 3.66 (m, 1H), 2.24 (dd, *J* = 12.9, 4.8 Hz, 1H), 1.94 – 1.83 (m, 1H), 0.96 (s, 3H). LRMS (ESI^+^) m/z: calcd for C_25_H_35_N_5_O_10_[M+Na]^+^, 445.3; found, 467.3；

**3.1.36 *(2S,3R,5S)-2-(4-aminopyrrolo[2,1-f][1,2,4]triazin-7-yl)-5-(hydroxymethyl)-3-methyltetrahydrofuran-3-ol* (CPD3c)**

To a solution of intermediate **22** (290 mg, 0.65 mmol, 1 eq) in acetic acid (5 mL) was added 10% Pd/C (135 mg, 0.13 mmol, 0.2 eq). The suspension was stirred under an atmosphere of H_2_ at 20 °C for 15 h. The reaction was filtered and concentrated to dryness. The residue was purified by flash column chromatography to yield **CPD3c** (51 mg, 29.7%) as white solid. ^1^H NMR (400 MHz, DMSO-*d*_6_) δ 7.81 (s, 1H), 7.62 (s, 3H), 6.83 (d, *J* = 4.4 Hz, 1H), 6.65 (d, *J* = 4.4 Hz, 1H), 5.34 (s, 1H), 5.06 (s, 1H), 4.76 (t, *J* = 5.6 Hz, 1H), 4.32 – 4.17 (m, 1H), 3.66 – 3.58 (m, 2H), 1.83 – 1.77 (m, 2H), 0.84 (s, 3H). ^13^C NMR (101 MHz, DMSO-*d*_6_) δ 155.6, 147.5, 131.3, 114.0, 108.9, 100.9, 82.3, 80.8, 79.0, 63.2, 41.7, 23.1. HR-ESI-MS: [M+H]^+^ calcd for C_12_H_17_N_4_O_3_, 265.1301, found 265.1295.

**3.1.37 *(3R,5S)-3-(benzyloxy)-5-((benzyloxy)methyl)-2-methoxytetrahydrofuran* (24)**

To a solution of **23** (5.0 g, 18.0 mmol, 1 eq) in methanol (50 mL) was added conc. H_2_SO4 (0.5 mL) at 0 °C. The solution was heated to 60°C and stirred for 3 h. After cooling to room temperature, NaHCO_3_ was added into the mixture and stirred for 30 min. The mixture was filtered to remove insoluble solid and the filtrate was concentrated to obtain a yellow oil, which was directly used in next step.

The oily residue was dissolved in methanol (50 mL). K_2_CO_3_ (2.4 g，18.0 mmol，1 eq) was added to the solution. After stirring at room temperature for 2 h, the mixture was filtered and the filtrate was concentrated to dryness. The residue was dissolved in THF (50 mL), and 60% NaH (1.4 g，36.0 mmol，2 eq) was added at 0 °C. After stirring for 10 min, benzyl bromide (6.0 g, 36 mmol, 2 eq) was added to the reaction. The mixture was stirred at room temperature for 6h, then slowly poured into saturated NH₄Cl aqueous solution. The mixture was extracted, washed, dried, concentrated and purified to obtained intermediate **24** (2.5 g, 42.3% yield over 3 steps) as colorless oil. ^1^H NMR (400 MHz, DMSO-*d*_6_) δ 7.37 – 7.28 (m, 10H), 4.91 (s, 1H), 4.54 – 4.51 (m, 4H), 4.38 – 4.30 (m, 1H), 3.93 (d, *J* = 5.0 Hz, 1H), 3.45 – 3.39 (m, 2H), 3.20 (s, 4H), 2.09 – 2.01 (m, 1H), 1.78 – 1.70 (m, 1H). LRMS (ESI+) m/z: calcd for C_20_H_24_O_4_ [M+Na]^+^, 328.1; found, 351.7

**3.1.38 *(3R,5S)-3-(benzyloxy)-5-((benzyloxy)methyl)tetrahydrofuran-2-ol* (25)**

Compound **24** (12.5 g, 38.11 mmol, 1 eq) was added into 80% AcOH in water (100 mL). The mixture was stirred at 100°C for 8 h, then cooled to room temperature. The mixture was concentrated under reduced pressure. The residue was purified by flash column to obtain **25** (8.3 g, 69.4%) as colorless oil. ^1^H NMR (400 MHz, DMSO-*d*_6_) δ 7.37 – 7.28 (m, 11H), 6.24 (d, *J* = 4.7 Hz, 1H), 5.21 (d, *J* = 4.8 Hz, 1H), 4.54 – 4.47 (m, 4H), 4.31 – 4.19 (m, 2H), 3.83 (d, *J* = 4.8 Hz, 1H), 3.53 – 3.43 (m, 2H), 2.06 – 2.00 (m, 1H), 1.85 – 1.73 (m, 1H). LRMS (ESI+) m/z: calcd for C_19_H_23_O_4_ [M+H]^+^, 315.4; found, 315.5;

**3.1.39** ***(3R,5S)-3-(benzyloxy)-5-((benzyloxy)methyl)dihydrofuran-2(3H)-one* (26)**

To a solution of **25** (8.3 g, 26.4 mmol, 1 eq) in DMSO (24 mL) was added acetic anhydride (16 mL) at room temperature. After stirring for 26 h, the mixture was poured into water, extracted with ethyl acetate, washed with water and brine, dried over anhydrous Na_2_SO_4_, concentrated to dryness, and purified by flash column chromatography to yield **26** (6.5 g, 78.9%) as colorless oil. ^1^H NMR (400 MHz, DMSO-*d*_6_) δ 7.37 – 7.25 (m, 10H), 4.82 – 4.78 (m, 1H), 4.76 (d, *J* = 12.0 Hz, 1H), 4.65 (d, *J* = 11.9 Hz, 1H), 4.50 (s, 2H), 4.40 (t, *J* = 7.8 Hz, 1H), 3.63 (dd, *J* = 11.0, 2.7 Hz, 1H), 3.54 (dd, *J* = 11.0, 4.0 Hz, 1H), 2.45 – 2.35 (m, 1H), 2.32 – 2.22 (m, 1H). LRMS (ESI^+^) m/z: calcd for C_19_H_21_O_4_ [M+H]^+^, 313.4; found, 313.5;

**3.1.40** ***7-((3R,5S)-3-(benzyloxy)-5-((benzyloxy)methyl)-2-methyltetrahydrofuran-2-yl)-7aH-cyclopenta[d]pyrimidin-4-amine* (28)**

Compound **27** (4.3 g, 46.3% yeild) was obtained from **26** (6.5 g, 20.8 mmol, 1 eq) and 7-iodopyrrolo[2,1-f][1,2,4]triazin-4-amine (5.3 g, 20.7 mmol, 1 eq) using the similar method with **21**.

To a solution of **27** (500 mg, 1.12 mmol, 1 eq) in anhydrous dichloromethane (5 mL) was added 46.5% boron trifluoride ether solution (1.37 g，4.49 mmol，4 eq) and 2.0 M AlMe_3_ (2.8 mL, 5.60 mmol, 5 eq) in at 0°C. After stirring at 0℃ for 4 h, the mixture was poured into saturated NaHCO_3_ aqueous solution, extracted with DCM, washed with water and brine, dried over anhydrous Na_2_SO_4_, concentrated to dryness, and purified by flash column chromatography to yield **28** (347 mg, 69.9% yeild) as colorless oil. ^1^H NMR (400 MHz, DMSO-*d*_6_) δ 7.75 (s, 1H), 7.56 (s, 2H), 7.39 – 7.35 (m, 5H), 7.12 – 7.05 (m, 2H), 6.80 (d, *J* = 4.3 Hz, 1H), 6.73 – 6.67 (m, 2H), 6.52 (d, *J* = 4.3 Hz, 1H), 4.57 (d, *J* = 12.6 Hz, 3H), 4.43 (d, *J* = 3.7 Hz, 1H), 4.39 (d, *J* = 11.6 Hz, 1H), 4.36 – 4.30 (m, 1H), 4.18 (d, *J* = 11.6 Hz, 1H), 3.63 (dd, *J* = 10.5, 4.0 Hz, 1H), 3.58 (dd, *J* = 10.2, 5.4 Hz, 1H), 2.17 (dd, *J* = 13.2, 5.6 Hz, 1H), 2.12 – 2.05 (m, 1H), 1.56 (s, 3H). LRMS (ESI^+^) m/z: calcd for C_25_H_35_N_5_O_10_[M+Na]^+^, 443.2; found, 467.3;

**3.1.41** ***(3R,5S)-2-(4-amino-7aH-cyclopenta[d]pyrimidin-7-yl)-5-(hydroxymethyl)-2-methyltetrahydrofuran-3-ol* (CPD3d)**

To a solution of intermediate **28** (340 mg, 0.77 mmol, 1 eq) in acetic acid (5 mL) was added 10% Pd/C (200 mg, 0.19 mmol, 0.25 eq). The suspension was stirred under an atmosphere of H_2_ at 20 °C for 24 h. The reaction was filtered and concentrated to dryness. The residue was purified by flash column chromatography to yield **CPD3d** (110 mg, 54.3% yeild) as anomeric mixture. ^1^H NMR (400 MHz, DMSO-*d*_6_) δ 7.76 (s, 1H), 7.50 (s, 2H), 6.77 (d, *J* = 4.2 Hz, 1H), 6.52 (d, *J* = 4.2 Hz, 1H), 4.69 (t, *J* = 5.9 Hz, 1H), 4.49 (d, *J* = 4.6 Hz, 1H), 4.41 (d, *J* = 4.6 Hz, 1H), 4.32 – 4.19 (m, 1H), 3.56 – 3.45 (m, 2H), 2.12 – 1.99 (m, 1H), 1.87 – 1.78 (m, 1H), 1.48 (s, 3H). ^13^C NMR (101 MHz, DMSO-*d*_6_) δ 155.5, 147.1, 133.9, 114.2, 108.6, 100.1, 85.8, 78.6, 76.2, 64.3, 36.8, 25.6. LRMS (ESI^+^) m/z: calcd for C_13_H_17_N_3_O_3_[M+23]^+^, 264.1; found, 265.3. HRMS (ESI^+^) m/z: calcd for C_13_H_18_N_3_O_3_[M+H]^+^, 264.1348; found, 265.1350.

**3.1.42 *(3aR,6R,6aR)-6-(((tert-butyldimethylsilyl)oxy)methyl)-4-(2,4-dichloro-pyrrolo[2,1-f][1,2,4]triazin-7-yl)-2,2-dimethyltetrahydrofuro[3,4-d][1,3]dioxol-4-ol* (31)**

Intermediate **29** (600 mg, 2.25 mmol, 1.5 eq) and **30** (452 mg, 1.50 mmol, 1 eq) was dissolved in anhydrous THF (10 mL). The solution was cooled to -78°C under N_2_ atmosphere, then 1.6 M n-butyllithium in hexanes (1.2 mL, 1.92 mmol, 1.2 eq) was dropwise added by syringe. After stirring at -78°C for 8 h, saturated NH_4_Cl aqueous solution was added into the solution, then the mixture was slowly warmed to room temperature. The mixture was extracted, washed, dried, concentrated and purified to obtained intermediate **31** (389 mg, 53.0% yeild) as yellow oil. ^1^H NMR (400 MHz, DMSO-*d*_6_) δ 7.22 (d, *J* = 4.8 Hz, 1H), 7.17 (d, *J* = 4.8 Hz, 1H), 7.08 (s, 1H), 5.05 (d, *J* = 6.0 Hz, 1H), 4.87 (dd, *J* = 5.8, 1.2 Hz, 1H), 4.21 – 4.14 (m, 1H), 3.79 – 3.70 (m, 2H), 1.18 (s, 3H), 1.09 (s, 3H), 0.90 (s, 9H), 0.10 (s, 6H). LRMS (ESI^+^) m/z: calcd for C_20_H_30_Cl_2_N_3_O_5_Si [M+H]^+^, 491.5; found, 491.4;

**3.1.43 *7-((3aS,6R,6aR)-6-(((tert-butyldimethylsilyl)oxy)methyl)-2,2-dimethyltetra hydrofuro[3,4-d][1,3]dioxol-4-yl)-2,4-dichloropyrrolo[2,1-f][1,2,4]triazine* (32)**

To a solution of **31** (1.2g, 2.45 mmol, 1 eq) in dichloromethane (4 mL) was added triethylsilane (1.14 g, 9.83 mmol, 4 eq) and 46.5% boron trifluoride ether solution (1.94 mL, 7.35 mmol, 3 eq) at 0°C. After stirring at 0°C for 1 h, the reaction mixture was poured into saturated aqueous NaHCO_3_ solution. The mixture was extracted with dichloromethane, washed with water and brine, dried over anhydrous Na_2_SO_4_, and concentrated to dryness. The residue was purified by flash column chromatography to yield **32** (487 mg, 42% yeild) as inseparable anomeric mixture (*α*：*β*=1:2). ^1^H NMR (400 MHz, DMSO-*d*_6_) δ 7.23 (d, *J* = 4.8 Hz, 1H), 7.13 (d, *J* = 4.8 Hz, 1H), 5.74 (d, *J* = 4.3 Hz, 1H), 5.02 (dd, *J* = 6.0, 4.3 Hz, 1H), 4.90 (d, *J* = 5.9 Hz, 1H), 4.23 (t, *J* = 3.6 Hz, 1H), 3.84 – 3.78 (m, 2H), 1.26 (s, 3H), 1.21 (s, 3H), 0.92 (s, 9H), 0.09 (s, 3H), 0.08 (s, 3H). LRMS (ESI^+^) m/z: calcd for C_20_H_30_Cl_2_N_3_O_4_Si [M+H]^+^, 475.5; found, 475.4;

**3.1.44 *(3R,4S,5R)-2-(2,4-dichloropyrrolo[2,1-f][1,2,4]triazin-7-yl)-5-(hydroxy-methyl)tetrahydrofuran-3,4-diol* (33a)**

A mixture of **32** (487 mg, 1.03 mmol, 1 equiv) and 7 N ammonia in methanol (5 mL) was added into a sealed tube and stirred at 80 °C for 16 h. After completion of the reaction, the solvent was removed under reduced pressure. The residue was purified by silica‑gel column chromatography to afford **33a** as a yellow oil (265 mg, 57.2% yield), obtained as an anomeric mixture (*α/β* = 3:2).

***α*-anomer**: ^1^H NMR (400 MHz, DMSO-*d*_6_) δ 8.32 – 8.25 (m, 2H), 6.93 (d, *J* = 4.5 Hz, 1H), 6.73 (d, *J* = 4.5 Hz, 1H), 5.22 (d, *J* = 4.2 Hz, 1H), 5.02 (dd, *J* = 6.5, 4.3 Hz, 1H), 4.73 (dd, *J* = 6.5, 3.6 Hz, 1H), 4.05 – 3.96 (m, 1H), 3.69 – 3.60 (m, 2H), 1.51 (s, 3H), 1.31 (s, 3H), 0.84 (s, 9H), -0.01 (s, 3H), -0.02 (s, 3H).

***β*-anomer**: ^1^H NMR (400 MHz, DMSO-*d*_6_) δ 8.19 (m, 2H), 6.93 (d, *J* = 4.5 Hz, 1H), 6.63 (d, *J* = 4.5 Hz, 1H), 5.60 (d, *J* = 4.0 Hz, 1H), 4.92 (dd, *J* = 6.0, 4.1 Hz, 1H), 4.85 (d, *J* = 6.0 Hz, 1H), 4.15 – 4.08 (m, 1H), 3.81 – 3.72 (m, 2H), 1.31 (s, 3H), 1.22 (s, 3H), 0.91 (s, 9H), 0.08 (s, 6H).

**3.1.45 *(3R,4S,5R)-2-(4-amino-2-chloropyrrolo[2,1-f][1,2,4]triazin-7-yl)-5-(hydroxymethyl)tetrahydrofuran-3,4-diol* (CPD3e)**

Compound **33a** (200 mg, 0.63 mmol, 1 eq) was added to 90% trifluoroacetic acid in water (4 mL). The mixture was stirred at room temperature for 2 h, then concentrated under reduced pressure. The residue was purified by flash column chromatography to yield a yellow oil, which then recrystallize in dichloromethane: methanol (1:1, *v: v*) to afford **CPD3e** (111 mg, 58.7% yeild) as white solid. ^1^H NMR (400 MHz, DMSO-*d*_6_) δ 8.28 – 8.19 (m, 2H), 6.93 (d, *J* = 4.5 Hz, 1H), 6.71 (d, *J* = 4.5 Hz, 1H), 5.05 (d, *J* = 6.7 Hz, 1H), 4.98 (d, *J* = 6.4 Hz, 1H), 4.92 (d, *J* = 5.2 Hz, 1H), 4.71 (t, *J* = 5.7 Hz, 1H), 4.26 – 4.16 (m, 1H), 3.99 – 3.91 (m, 1H), 3.83 – 3.75 (m, 1H), 3.61 – 3.48 (m, 1H), 3.47 – 3.40 (m, 1H). ^13^C NMR (101 MHz, DMSO-*d*_6_) δ 155.8, 152.2, 130.2, 112.7, 110.4, 102.3, 84.7, 74.7, 73.9, 71.3, 62.0. HRMS (ESI^+^) m/z: calcd for: C_11_H_14_ClN_4_O_4_ [M+H]^+^, 301.0704; found, 301.0595.

**3.1.46 *(3R,4S,5R)-2-(2-chloro-4-((2-chlorobenzyl)amino)pyrrolo[2,1-f][1,2,4] triazin-7-yl)-5-(hydroxymethyl)tetrahydrofuran-3,4-diol* (CPD3f)**

Compound **32** (400 mg, 1.25 mmol, 1 eq), 2-chlorobenzylamine (355 mg, 2.50 mmol, 2 eq) and triethylamine (378 mg, 3.75 mmol, 3 eq) was added to ethanol (5 mL). The mixture was stirred at 50 °C for 10 h, then concentrated to dryness. The residue was partitioned between ethyl acetate and water, then the organic layer was collected, washed, dried and concentrated to dryness. The obtained yellow oil was added to 90% trifluoroacetic acid in water (5 mL). The mixture was stirred at room temperature for 2 h, then concentrated under reduced pressure. The residue was purified by flash column chromatography to yield **CPD3f** (427 mg, 80.4% yeild) as white foam. ^1^H NMR (400 MHz, DMSO-*d*_6_) δ 9.16 (t, *J* = 5.7 Hz, 1H), 7.52 – 7.46 (m, 1H), 7.41 – 7.29 (m, 3H), 7.05 (d, *J* = 4.6 Hz, 1H), 6.68 (d, *J* = 4.5 Hz, 1H), 4.92 – 4.87 (m, 2H), 4.78 (dd, *J* = 5.7, 3.8 Hz, 2H), 4.73 (d, *J* = 6.6 Hz, 1H), 4.55 (d, *J* = 7.7 Hz, 1H), 4.02 – 3.96 (m, 1H), 3.85 – 3.77 (m, 1H), 3.65 – 3.58 (m, 1H), 3.56 – 3.50 (m, 1H), 3.50 – 3.44 (m, 1H). ^13^C NMR (101 MHz, DMSO-*d*_6_) δ 154.1, 151.7, 135.3, 132.3, 130.7, 129.4, 129.1, 127.4, 112.4, 110.4, 102.0, 71.1, 69.6, 67.2, 67.0, 65.4. HRMS (ESI^+^) m/z: calcd for: C_18_H_19_Cl_2_N_4_O_4_ [M+H]^+^, 425.0783; found, 425.0790.

**3.1.47 *(2S,3R,5S)-2-(4-amino-2-chloropyrrolo[2,1-f][1,2,4]triazin-7-yl)-5-(hydroxymethyl)tetrahydrofuran-3-ol* (CPD3g)**

**CPD3g** (white solid, 61 mg, 21.5% overall yield) was obtained from **CPD3e** (300 mg, 1.0 mmol, 1 eq) using the **General procedure C**. ^1^H NMR (400 MHz, DMSO-*d*_6_) δ 8.25 – 8.17 (m, 2H), 6.91 (d, *J* = 4.4 Hz, 1H), 6.66 (d, *J* = 4.3 Hz, 1H), 5.23 (d, *J* = 4.5 Hz, 1H), 5.04 (d, *J* = 3.3 Hz, 1H), 4.69 (t, *J* = 5.9 Hz, 1H), 4.39 – 4.32 (m, 1H), 4.22 – 4.14 (m, 1H), 3.54 – 3.42 (m, 2H), 2.00 – 1.89 (m, 1H), 1.88 – 1.78 (m, 1H). ^13^C NMR (101 MHz, DMSO-*d*_6_) δ 156.2, 152.6, 131.5, 112.9, 110.4, 102.8, 79.7, 79.6, 74.8, 64.2, 37.3. HRMS (ESI^+^) m/z: calcd for: C_11_H_14_ClN_4_O_3_ [M+H]^+^, 285.0754; found, 285.0773.

**References**

1.	Martínez‐Montero S, Fernández S, Rodríguez‐Pérez T, et al. Improved Synthesis and Isolation of 2′‐ O ‐Methyladenosine: Effective and Scalable Enzymatic Separation of 2′/3′‐ O ‐Methyladenosine Regioisomers. *Eur J Org Chem*. 2009;2009(19):3265-3271.

2.	Gosselin, Gilles; Bergogne, Marie Christine; De Rudder, Jean; De Clercq, Erik; Imbach, Jean Louis . (1987). Systematic synthesis and biological evaluation of .alpha.- and .beta.-D-lyxofuranosyl nucleosides of the five naturally occurring nucleic acid bases. Journal of Medicinal Chemistry, 30(6), 982–991.

3.	Mort CJW, Migaud ME, Galione A, Potter BVL. Aplysia californica mediated cyclisation of novel 3'-modified NAD+ analogues: a role for hydrogen bonding in the recognition of cyclic adenosine 5'-diphosphate ribose. *Bioorg Med Chem*. 2004;12(2):475-487.

4.	Eldrup AB, Allerson CR, Bennett CF, et al. Structure-activity relationship of purine ribonucleosides for inhibition of hepatitis C virus RNA-dependent RNA polymerase. *J Med Chem*. 2004;47(9):2283-2295.

5.	Hulpia F, Balzarini J, Schols D, Andrei G, Snoeck R, van Calenbergh S. Exploring the purine core of 3'-C-ethynyladenosine (EAdo) in search of novel nucleoside therapeutics. *Bioorg Med Chem Lett*. 2016;26(8):1970-1972.

6.	Wang G, Deval J, Hong J, et al. Discovery of 4'-chloromethyl-2'-deoxy-3',5'-di-O-isobutyryl-2'-fluorocytidine (ALS-8176), a first-in-class RSV polymerase inhibitor for treatment of human respiratory syncytial virus infection. *J Med Chem*. 2015;58(4):1862-1878.

7.	Clarke MO, Mackman R, Byun D, et al. Discovery of β-D-2'-deoxy-2'-α-fluoro-4'-α-cyano-5-aza-7,9-dideaza adenosine as a potent nucleoside inhibitor of respiratory syncytial virus with excellent selectivity over mitochondrial RNA and DNA polymerases. *Bioorg Med Chem Lett*. 2015;25(12):2484-2487.

8.	Bouton J, Maes L, Karalic I, Caljon G, van Calenbergh S. Synthesis and evaluation of a collection of purine-like C-nucleosides as antikinetoplastid agents. *Eur J Med Chem*. 2021;212:113101.

9.	Metobo SE, Xu J, Saunders OL, et al. Practical synthesis of 1′-substituted Tubercidin C-nucleoside analogs. *Tetrahedron Letters*. 2012;53(5):484-486.

10.	Vodnala SK, Lundbäck T, Yeheskieli E, et al. Structure-activity relationships of synthetic cordycepin analogues as experimental therapeutics for African trypanosomiasis. *J Med Chem*. 2013;56(24):9861-9873.

11.	WO_2010081082_A2.

## 3.2. ^1^H NMR and ^13^C NMR of the compounds

**Figure S8.** ^1^H-NMR and ^13^C NMR spectrum of **CPD1a**

**Figure S9.** ^1^H-NMR and ^13^C NMR spectrum of **CPD1b**

**Figure S10.** ^1^H-NMR and ^13^C NMR spectrum of **CPD1c**

**Figure S11.** ^1^H-NMR and ^13^C NMR spectrum of **CPD1d**

**Figure S12.** ^1^H-NMR and ^13^C NMR spectrum of **CPD1e**

**Figure S13.** ^1^H-NMR spectrum of **1**

**Figure S14.** ^1^H-NMR spectrum of **2**

**Figure S15.** ^1^H-NMR spectrum of **3**

**Figure S16.** ^1^H-NMR spectrum of **4**

**Figure S17.** ^1^H-NMR and ^13^C NMR spectrum of **CPD1f**

**Figure S18.** ^1^H-NMR spectrum of **5**

**Figure S19.** ^1^H-NMR spectrum of **6**

**Figure S20.** ^1^H-NMR spectrum of **7**

**Figure S21.** ^1^H-NMR spectrum of **8b**

**Figure S22.** ^1^H-NMR spectrum of **8d**

**Figure S23.** ^1^H-NMR spectrum of **CPD1g**

**Figure S24.** ^1^H-NMR spectrum of **CPD1h**

**Figure S25.** ^1^H-NMR spectrum of **CPD1i**

**Figure S26.** ^1^H-NMR and ^13^C-NMR spectrum of **CPD1j**

**Figure S27.** ^1^H-NMR spectrum of **10a**

**Figure S28.** ^1^H-NMR spectrum of **10b**

**Figure S29.** ^1^H-NMR and ^13^C-NMR spectrum of **CPD2a**

**Figure S30.** ^1^H-NMR and ^13^C-NMR spectrum of **CPD2b**

**Figure S31.** ^1^H-NMR and ^13^C-NMR spectrum of **CPD2c**

**Figure S32.** ^1^H-NMR and ^13^C-NMR spectrum of **CPD2d**

**Figure S33.** ^1^H-NMR and ^13^C-NMR spectrum of **CPD2e**

**Figure S34.** ^1^H-NMR spectrum of **13a**

**Figure S35.** ^1^H-NMR and ^13^C-NMR spectrum of **CPD3a**

**Figure S36.** ^1^H-NMR and ^13^C-NMR spectrum of **CPD3b**

**Figure S37.** ^1^H-NMR and ^13^C-NMR spectrum of **CPD3c**

**Figure S38.** ^1^H-NMR and ^13^C-NMR spectrum of **CPD3d**

**Figure S39.** ^1^H-NMR spectrum of **31**

**Figure S40.** ^1^H-NMR spectrum of **32**

**Figure S41.** ^1^H-NMR spectrum of **33a** (α- and β-anomers)

**Figure S42.** ^1^H-NMR and ^13^C-NMR spectrum of **CPD3e**

**Figure S43.** ^1^H-NMR and ^13^C-NMR spectrum of **CPD3f**

**Figure S44.** ^1^H-NMR and ^13^C-NMR spectrum of **CPD3g**
